# A New Approach to Large Multiomics Data Integration

**DOI:** 10.1021/acs.analchem.5c01812

**Published:** 2025-09-11

**Authors:** Alex Dexter, Spencer A. Thomas, Rory T. Steven, Kenneth N. Robinson, Adam J. Taylor, Efstathios A. Elia, Chelsea Nikula, Andrew D. Campbell, Yulia Panina, Arafath K. Najumudeen, Bin Yan, Piotr Grabowski, Gregory Hamm, John Swales, Aurelien Tripp, George Poulogiannis, Mariia O. Yuneva, Simon Barry, Richard J.A. Goodwin, Owen J. Sansom, Zoltan Takats, Josephine Bunch

**Affiliations:** † 9917 National Physical Laboratory, Teddington TW11 0LW, U.K.; ‡ 722802 Bactobio, London SE11 5JH, U.K.; § 487206 Sage Bionetworks, Seattle, Washington 98121-1031, United States; ∥ Department of Chemistry, 54557 University of Cyprus, Nicosia 2109, Cyprus; ⊥ 3532 Beatson Cancer Research UK Institute, Glasgow G12 0YN, U.K.; # 376570 The Francis Crick Institute, London NW1 1AT, U.K.; ∇ Institute for Molecular Medicine Finland (FIMM) HiLIFE, 168536 University of Helsinki, Helsinki 00290, Finland; ○ 26239 Biological Insights Knowledge Graph, AI Strategy and Innovation, R&D IT, AstraZeneca, Barcelona 08028, Spain; ◆ Integrated Bioanalysis, Clinical Pharmacology & Safety Sciences, R&D, AstraZeneca, Cambridge CB2 0AA, U.K.; ¶ 5053 The Institute of Cancer Research, London SW3 6JB, U.K.; ⧅ Department of Metabolism, Digestion and Reproduction, 4615 Imperial College London, London W12 0NN, U.K.

## Abstract

Data reduction and data mining are common practices for handling large-scale data from wide-ranging sources, but high-dimensional omics and imaging data sets present difficult challenges for feature extraction and data mining due to the large number of features that cannot be simultaneously examined. The sample numbers and variables in these methods are constantly growing as new technologies are developed, and computational analysis needs to evolve to keep up with growing demand. In recent years, there has been a rapid uptake of nonlinear dimensionality reduction via methods such as t-distributed stochastic neighbor embedding and uniform manifold approximation and projection. These approaches have revolutionized our ability to visualize and interpret high-dimensional data and have rapidly become preferred methods for analysis of data sets containing an extremely high number of variables. Further to this is the emerging interest in combining information from multiple omics sources to gain a more holistic view of systems biology. Current state-of-the-art algorithms can perform data mining, visualization, and classification on routine data sets but struggle when data sets grow above a certain size. We present a new approach to large and multiomic data integration to extract, mine, and integrate large multiomics data sets that were previously considered prohibitively large. Here, we demonstrate the use of deep learning on subsampled nonlinear dimensionality reduction using t-SNE and UMAP to extract features from large complex data sets including mass spectrometry imaging and chromosome conformation capture. We then go on to demonstrate how this method can be used to learn embeddings from the fusion of different omics data, allowing metabolomics data to be projected into a reduced transcriptomics representation.

## Graphical abstract

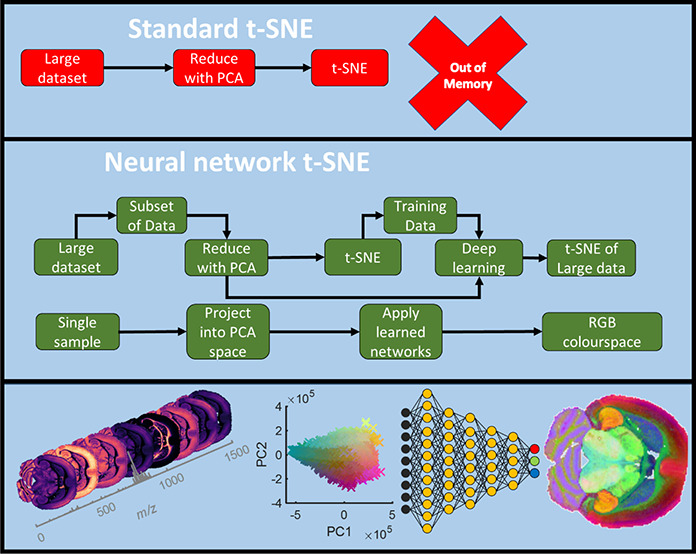

## Introduction

Since the introduction of genomics in 1987 the field of “omics”, including data from transcriptomics, proteomics, and metabolomics,^1,2^ has evolved rapidly. These are now widely used in both preclinical and clinical settings to answer complex biological questions.^3^ There are three emerging themes within omics analysis: single cell omics,^4^ spatial omics,^5^ and multiomics integration.^6,7^ While these have presented novel opportunities for studying complex diseases, they have also created new challenges in integrative analysis, when considering the size and complexity of the resulting data. An unmet challenge in large and multiomics studies remains the handling, visualization, and interpretation of the complex data.

When describing big data, two types of big data challenges are often discussed; data with large sample numbers such as stock exchange, marketing, and social media data, and data that contains a large variable space (high dimensionality), such as genomics, transcriptomics, and mass spectrometry-based omics (proteomics and metabolomics). There is an increase in data that are large in both samples and variables such as spatial and single cell omics data.^8^ In addition to this, the integration of multiple omics data is an increasingly important topic, as this enables a more complete understanding of disease complexity to be obtained. For example Chaudhary et al. recently used a deep learning multiomics approach to determine features from three different sequencing data types (RNA sequencing, miRNA sequencing, and methylation) associated with differential survival of hepatocellular carcinoma.^6^ This can be achieved by concatenating and performing the reduction of these integrated data,^6^ through network-based approaches,^7^ factorization,^9^ or Bayesian^10^ methods. These different methods typically aim to create a joint embedding of different omics data, which then acts as the starting point for data mining and interpretation.

One other challenge in high dimensional omics data are the nonlinear relationships between variables. Nonlinear dimensionality reduction methods such as t-distributed stochastic neighbor embedding (t-SNE) and uniform manifold approximation projection (UMAP) have been shown to outperform linear methods such as principal component analysis (PCA) in all omics fields.^11^ Nonlinear dimensionality reduction of a single omics data, and multiomics data integration both suffer from the additional challenge of computational complexity. The algorithms developed for these methods while powerful, cannot be readily applied to very large complex data sets such as large cohort studies.

When data sets are particularly large, many of these algorithms fail due to lack of memory (RAM), and in extreme cases even loading the full data set into memory becomes prohibitive. Examples of these include large Hi-C data, single cell transcriptomics, and mass spectrometry imaging (MSI). Abdelmoula et al. recently demonstrated the use of a hierarchical stochastic neighbor embedding method using landmark features to embed and visualize large 3D mass spectrometry imaging data sets.^12^ This allowed t-SNE dimensionality reduction to be performed on data sets with over 1 million pixels such as 3D MSI data. Along with this, Boytsov et al. recently proposed the use of local interpolation with outlier control t-SNE (LION-tSNE) to embed newly acquired data into previously t-SNE mapped space.^13,14^ As well as allowing t-SNE to be performed on large data sets, this also allows new data to be incorporated into the embedded 2D or 3D space. These demonstrate the growing need for scalable nonlinear embedding algorithms for omics data.

In comparison to single omics reduction, most of the multiomics data integration methods are focused on linear methods such as non-negative matrix factorization.^9^ This can be attributed to the additional computational complexity of integration tasks, as well as a lack of direct model to more effectively relate input and output variables. Deep learning via neural networks are regularly used to learn mathematical transformations to go from high to low dimensional space in a nonlinear manner. These can be either supervised such as those used in classification problems,^15^ or unsupervised, such as stacked and variational autoencoders.^16^ Neural networks are often applied in classification problems, demonstrating greater accuracy than linear approaches.^17^ Recently, there has been a development of methods using neural networks and autoencoders to perform dimensionality reduction itself. In particular, methods such as parametric t-SNE, VAE-SNE and net-SNE have used the Kullback–Leibler divergence used in t-SNE as an optimization function for autoencoders to produce similar results to t-SNE.^18^ Other methods such as IVIS use Siamese neural networks with triplet loss function to preserve local and global similarity in the reduced space.^19^ Recently Abdelmoula et al. demonstrated the usefulness of these types of approaches to return spectral information from MSI data, as well as their capability to handle large data sets.^20^

To date, the majority of developments in neural network based nonlinear reduction have aimed at performing the nonlinear reduction optimization using cost functions derived from existing methods such as t-SNE. Espadoto et al. showed that neural networks can be trained on t-SNE and UMAP projections directly, and demonstrated this on relatively small testing data sets.^21^ Here, we demonstrate the use of deep learning on subsampled nonlinear dimensionality reduction using t-SNE and UMAP to extract features from large complex biological data sets. We then go on to demonstrate how this method can be used to learn embeddings from the fusion of different omics data, allowing metabolomics data to be projected into a reduced transcriptomics representation. This can be used to perform nonlinear multiomics integration and large-scale data analysis in an efficient manner.

## Materials and Methods

### Data Processing

Data processing was performed on an Intel Xeon quad core CPU E5–2680 v3 (2.50 GHz) ×2 with 128 GB of RAM and for the subset comparison, on an Intel Xeon CPU E5-2698 v4 (2.2 GHz) ×2 with 1 TB of RAM. Data were converted from the proprietary Waters.RAW format into imzML using ProteoWizard^22^ and imzML converter,^23^ and imported into Matlab (version 2017a and statistics and image processing toolbox, The Math-Works, Inc., Natick, MA, USA) using SpectralAnalysis.^24^ Ion images were generated by integrating intensities across each peak. Mean spectra were generated after preprocessing with interpolated rebinning using a bin width of 0.002 Da.^24^ Once imported into Matlab data subsets were taken by selecting every *n*th pixel where *n* is specified by the user (based on memory and time constraints). For nonlinear dimensionality reduction we have used t-SNE and UMAP as exemplar methods as they are the two most commonly used in many omics fields, but the principles of the approach are applicable to any nonlinear dimensionality reduction method. t-SNE was performed with the Matlab function “tsne” (Statistics toolbox) using the exact algorithm, perplexity of 30, exaggeration of 4, and correlation distance metric.^25^ UMAP was performed using the code provided by Meehan et al. *(* https://www.mathworks.com/matlabcentral/fileexchange/71902, accessed 25/02/2020) using the default parameters (30 nearest neighbors, and minimum distance of 0.3), apart from correlation distance metric and reduction to three dimensions.

Neural network training was performed using the Matlab neural network training tool (Neural network toolbox), using 70% training data, 15% testing data, and 15% validation data, with 1000 max epochs, and Bayesian regularization as the training method unless otherwise stated.

### Hi-C Data

Combined inter- and intrachromosomal contact matrices of human GM12878 cell line generated by (8) were downloaded from the Gene Expression Omnibus (GEO) using the GSE63525 identifier. The contact matrices used in this study were of 1 kb resolution, un-normalized (.RAWobserved files), reads mapping to the genome with MAPQ score >0. The mitochondrial and sex determination chromosomes (X and Y) were not used in this analysis. Data were loaded in sequentially additively binned into 50 kb columns (1 kb rows were still retained), and any fully zero rows or columns were removed. Training data was taken every 1000th sample resulting in a total of 2,882 samples. The A/B subcompartment annotation file for GM12878 cell line was downloaded from the same GEO repository (file GSE63525_GM12878_subcompartments.bed.gz). The H3K27ac ChIP-seq data for the GM12878 cell line were obtained from the ENCODE consortium.^26^ The fold-change over control signal from two biological replicates was used (file ENCFF180LKW.bigWig). Parsing of bigWig files and annotation of genomic elements was performed using R packages rtracklayer 1.48 and GenomicRanges 1.40.

### GEMM Colon Models

All animal experiments were carried out in accordance with UK Home Office regulations (PPL 70/8646), and subject to ethical review by the animal welfare and ethical review board of the University of Glasgow. The genetic alleles used in this study were *Vil* ^CreERT2^ (El-Marjou et al),^42^ *Apc* ^fl^ (Shibata et al. 1997),^43^ *Kras* ^G12D^ (Jackson et al. 2001)^44^ and *Pten* ^fl^ (Suzuki et al. 2001).^45^ All experiments were carried out in 8–12 week old mice of a pure, inbred C57BL6/J genetic background. Cre-mediated genetic recombination was induced through a single intraperitoneal injection of tamoxifen (80 mgkg^–1^) on one occasion (*Vil* ^CreERT2^ *Apc* ^fl/fl^ *Kras* ^G12D/+^ or *Vil* ^CreERT2^ *Apc* ^fl/fl^ *Kras* ^G12D/+^ *Pten* ^fl/fl^), or two consecutive days (*Vil* ^CreERT2^, *Vil* ^CreERT2^ *Apc* ^fl/fl^, *Vil* ^CreERT2^ *Apc* ^fl/fl^ *Pten* ^fl/fl^ or *Vil* ^CreERT2^ *Kras* ^G12D/+^), with animals sacrificed and tissues harvested at 72 or 96 h post induction, respectively. For subsequent analysis by MSI, whole snap-frozen tissue specimens were embedded in a 12.5% (w/v) solution of carboxymethylcellulose prior to sectioning. For REIMS analysis, intestinal epithelial cells were extracted from the small intestine or colon of these genetically engineered mouse models as described previously (Marsh 2008),^46^ snap frozen and stored in Eppendorf tubes at −80 °C until analysis.

### MSI Analysis

MALDI MSI analysis of the breast and colorectal cancer and sagittal, and coronal mouse brain samples, were acquired on Waters Synapt G2si Q-ToF instruments (Waters, UK) with a MALDI source for the breast and colorectal cancer samples, and a prototype uMALDI source for the sagittal mouse brain samples. The transverse mouse brain data were acquired on a Waters Xevo G2-XS Q-ToF instrument with a DESI ion source (Waters, UK) and the glioblastoma samples were acquired using a Bruker RapiFlex instrument (Bruker, Bremen, Germany). A full summary of experimental prarmeters for MSI data is provided in Table S1.

### REIMS Analysis

REIMS of the colon tissue cell extracts was performed using a Waters Xevo G2-XS (Waters, UK), operated in negative ion mode, 50–1500 *m*/*z* at 2 scans/s, using commercially available bipolar forceps for sample mobilization. An Erbe VIO 50C electrosurgical generator (Erbe Elektromedizin, Germany) was operated in bipolar mode at an output power of 15 W. The aerosol generated from the sample was aspirated and transferred to the REIMS source using Tygon tubing where it was mixed with propan-2-ol in a “t-piece” prior to introduction to the mass spectrometer. REIMS data from the breast tissue cell extracts was performed with commercially available electrosurgical bipolar forceps (Erbe Elektromedizin, Germany) connected to a ForceTriad electrosurgical unit (Covidien, Ireland) programmed in Macro bipolar setting using 4 W power for cell lines. Bipolar forceps were connected to the inlet capillary of a Thermo Exactive orbitrap instrument (Thermo Scientific) using PTFE tubing, allowing for the direct suction of aerosol generated from rapid biomass heating to the mass spectrometer.

### Description of the Method

Our approach is to take a subset of the data and perform nonlinear dimensionality reduction on it. The resulting dimensionally reduced data is then used as the training set for deep learning using neural networks. The remaining data not used for training is then embedded into the same reduced space along with any other new data subsequently measured. A similar set of networks are also trained on the reverse order from lower into higher dimensional space (Figure 1). This allows any hypothetical point in the low dimensional space to be returned into a predicted high dimensional data point. Moreover, neural networks are trained to reduce data from one omics (metabolomics) into the reduced space of another (transcriptomics) for matched samples. This algorithm includes several steps, each of which could be independently optimized, and many will not be independent of one another. A fully exhaustive comparison of these parameters is not feasible in the current work, but we will discuss the main parameters that are unique to this method. These include subset size used, method of subsampling the data, and the algorithm used when training the neural networks themselves. In all other cases, optimal parameters taken from prior literature have been used. A detailed summary of parameter optimization can be found in the Supporting Information.

**Figure 1:**
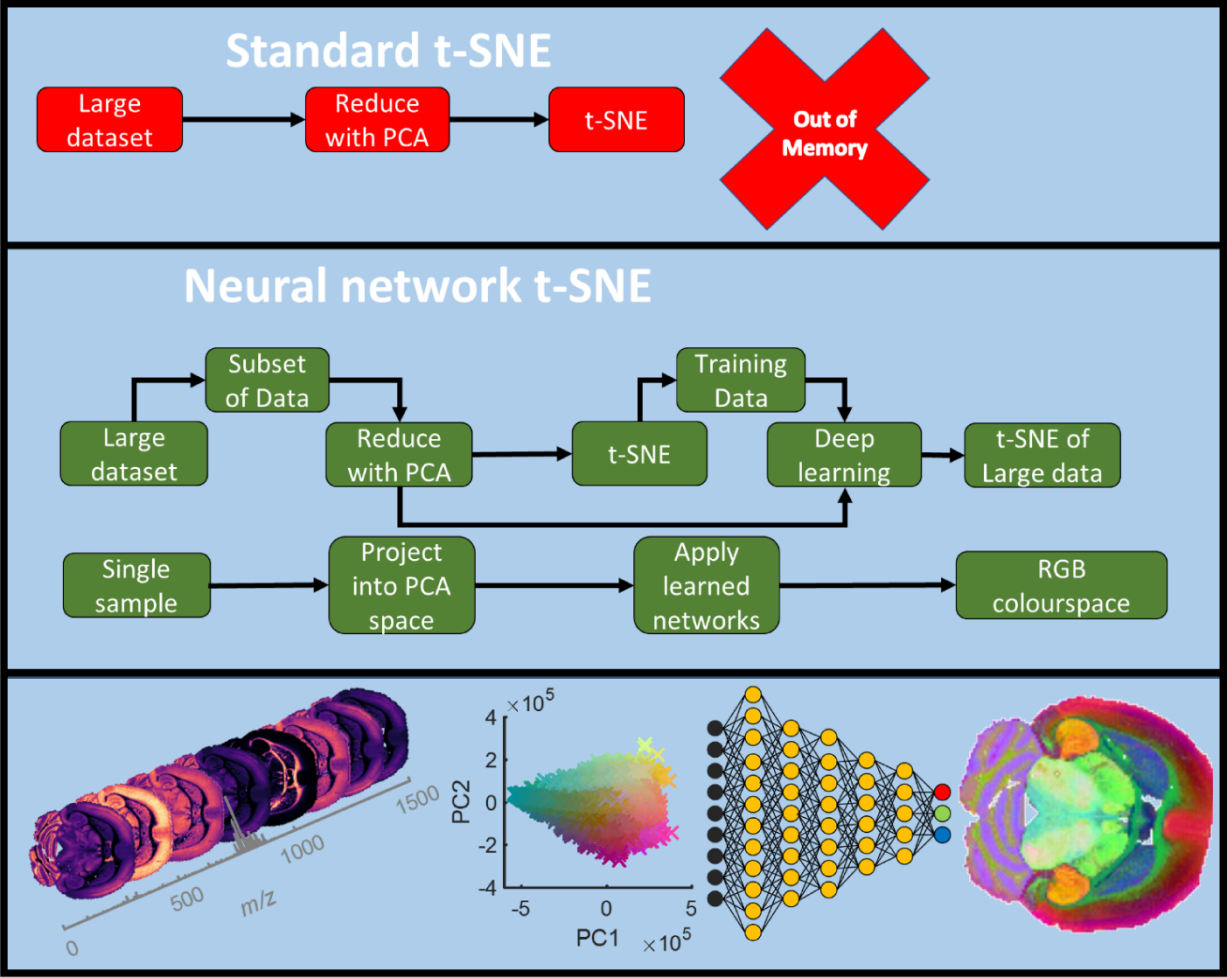
Outline of the novel workflow using t-SNE as the base dimensionality reduction method. A large data set that cannot have t-SNE performed on it is subsampled, and t-SNE is performed on the subset of data. The resulting embedding is then used as training data to train deep learning via neural networks to go from the high to low dimensional space. The networks can then be applied to remaining data (and any new data) resulting in reduction of the full data set into an approximation of t-SNE.

## Results

### Example Case Studies

#### Returning Spectral Information Using MSI Data

One of the biggest limitations of nonlinear methods such as t-SNE and UMAP, as compared to other dimensionality reduction methods like PCA and non-negative matrix factorization is that no spectral contributions driving the differences between features can be obtained. This is critical in omics studies where these can often be translated directly into disease biomarkers and potential drug targets.^2^ Unlike the challenges of scaling and addition of new data, these have yet to be fully addressed. In the our method, by training the neural networks to go from low to high in addition to high to low dimensional space, we can obtain a transformation to go from any three-dimensional point into a mass spectrum, and thus potential molecules. By performing this for red ([1 0 0]) green ([0 1 0]) and blue ([0 0 1]) we can obtain the relative spectral contributions along these dimensions to our image segmentation. Alternatively, we can segment the reduced space by methods such as clustering, and calculate centroids in the reduced space to determine the spectrum that would be derived from that region. We demonstrate this capability using desorption electrospray ionization DESI MSI data of mouse mammary gland tumors containing 90,000 samples and 2,000 variables.

Mammary gland tumors were generated in transgenic mice following ectopic expression of either MYC or ErbB2 under the control of the mouse mammary tumor virus (MMTV) promoter,^27^ and normal mammary glands (NMG) from healthy mice were also included in the MSI analysis as a control. The oncogene MYC is amplified in approximately 15% of breast cancers, it is associated with poor prognosis and correlates with unique metabolic vulnerabilities.^28^ ErbB2/HER2 is overexpressed in 20% of human breast cancers and correlates with tumor chemo-resistance and poor prognosis.^29^ Consistent with previous data to show that these two oncogenes are associated with distinct metabolic profiles,^30^ NN t-SNE of MSI data following background subtraction (Figure 2) clearly segments MYC from ErbB2 tumors and NMG samples in low dimensional space (Figure 2a,b). Bounding boxes describe distinct areas including leached fatty regions from the NMG, and the majority of pixels in the MYC or ErbB2 tumors (Figure 2c).

**Figure 2:**
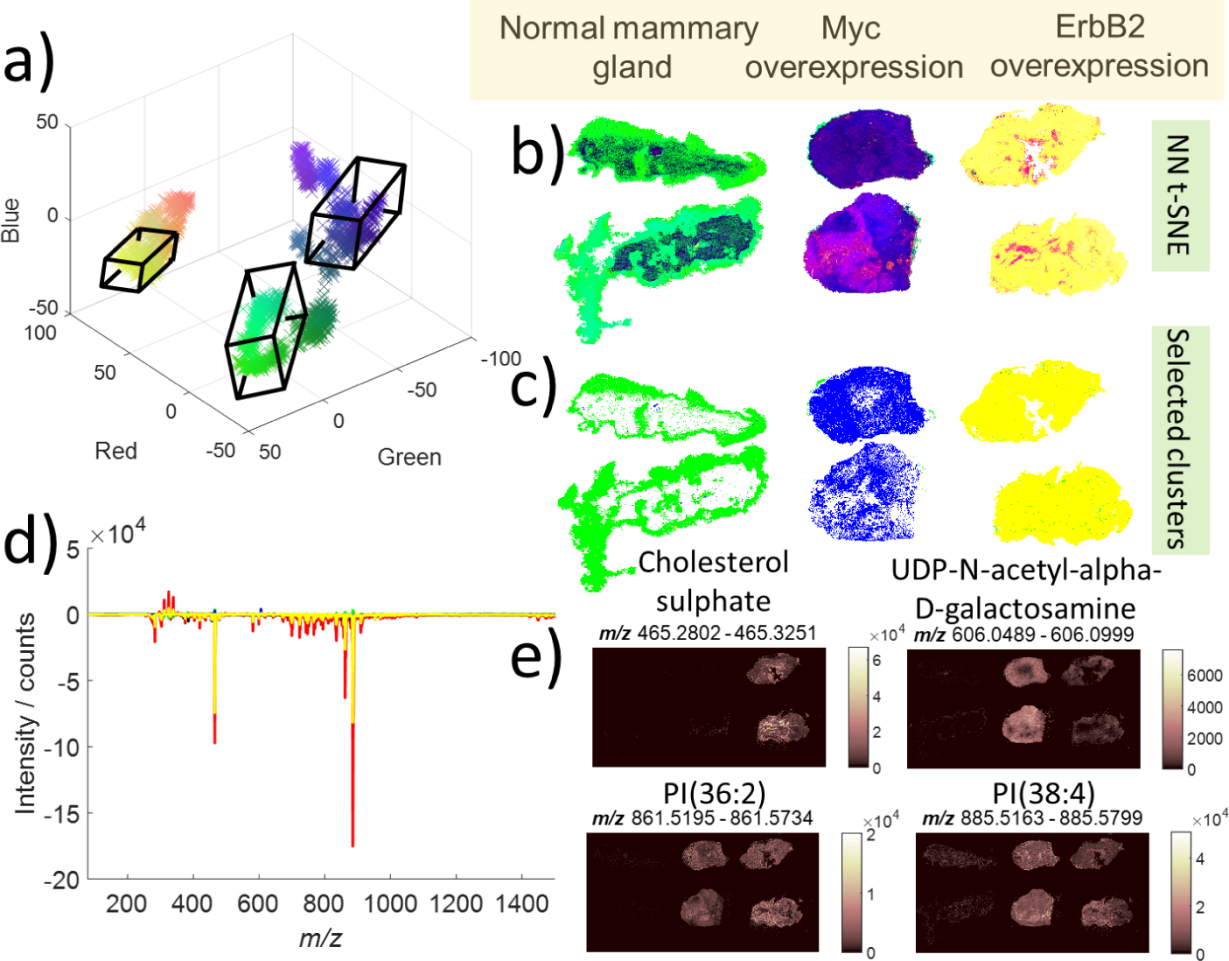
Results of NN t-SNE applied to MSI data from genetically engineered breast cancer models. The three genetic models are clearly differentiated in both the scatter plot (a) and image (b). These can be segmented to give masks for the different models (c), and the underlying spectral drivers can be identified (d) and their corresponding ion images generated (e).

While such spatial segmentation is useful, in order to provide biological interpretation, we must interrogate which spectral features are driving the separation in low dimensional space. We generated the spectral representation for selected corners of the low dimension space (red, green, blue, and yellow) (Figure 2d). Spectral representations may include positive and negative values indicating their relative contribution at the selected coordinates. Selected peaks with strong contributions to the spectra representation for each selected low dimensional coordinate were matched by exact mass to HMDB-v4.0 database^31^ and putative annotations made (Figure 2e). Cholesterol sulfate, a structural component of cellular membranes, has been previously identified in DESI MS experiments as a potential biomarker of prostate cancer.^32^ Here it is a strong contributor to the “yellow” corner of low dimensional space where pixels from the ErbB2 tumor are positioned. This appears to be a strong marker for ErbB2 over MYC tumors and NMG and it is localized across the whole tumor. Two peaks annotated as PI lipids are prominent in the spectral representation of the red and yellow side of low dimensional space. In the single ion images, they are localized in the MYC and ErbB2 tumors but absent in the NMG. As cell density is notably higher in the tumors than the sparse fat pad, this elevation of structural lipids in the tumors is expected. A notable contributor to the spectral representation of the “blue” corner of low dimension space is *m*/*z* 606.076, assigned as UDP-N-actyl-alpha-d-galactosamine. This nucleotide sugar has been implicated in protein glycosylation,^33^ which is frequently altered across all stages of tumor progression.^34^ It was detected in both tumor types, but was significantly more elevated in MYC-driven tumors, with some heterogeneity within individual tumors. This analysis not only provides nonlinear segmentation of spatial metabolomics data, but also assists with the determination of driving contributors to this segmentation, which can provide biological insights of genetically distinct subtypes, leading to potential new biomarkers and drug targets.

#### Addition of New Data

Any requirement to incorporate new data into embedding via t-SNE would necessitate rerunning the whole process to obtain unified segmentation of all data. This would be time-consuming, may produce a different color mapping (in the case of stochastic methods), and may exceed the memory constraints of this algorithm. Using the our method, new data can be embedded into the same low dimensional space of the previously segmented data. This can either be used to find data similar to the existing data, or data that is different from the training data for outlier detection.

We demonstrate the addition of new data on 3D MSI data of mouse brain tissue sections bearing glioblastoma. First, data from one of the tissue sections was used as the training data for the t-SNE algorithm, and neural networks were used to learn the mathematical transformations to go from the original to the t-SNE space. The trained network was then applied to the data from the remaining 29 serial sections, and a consistent segmentation of the same anatomical features into the same colors was observed (Figure 3a). As well as producing consistent segmentation of anatomy, the application of the network to the new data took around 1.5 s per data set, far faster than the data acquisition itself. The consistent segmentation provided by our method is an excellent platform for registration and 3D segmented image generation (Figure 3b).

**Figure 3:**
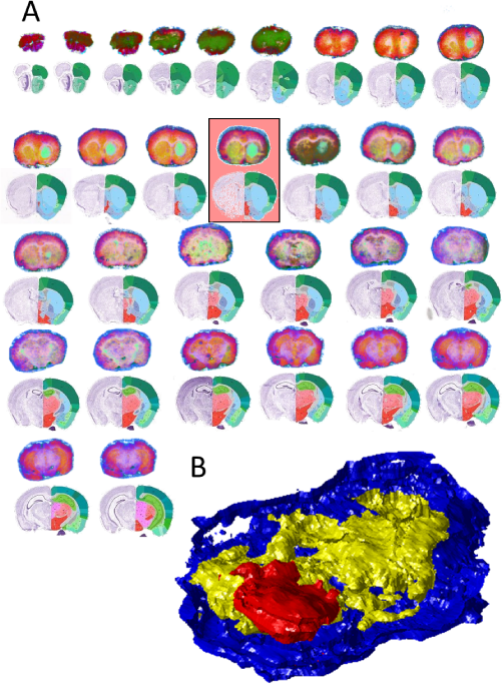
Results of segmentation using neural network t-SNE trained on MSI data from a single section of murine brain (highlighted in red) containing glioblastoma (segmented in light green). The data from the remaining sections were then reduced using the trained networks to segment the same anatomies in the same color scheme. By embedding data into the same color space similar features can easily be identified, and the results of this can easily be used to perform registration to generate 3D representations such as shown in (B).

In addition to consistently segmenting the same anatomies of similar data, new data that is dissimilar to all existing data could be identified. This is demonstrated on the same MSI data from the serial mouse brain sections. By training the network on t-SNE applied to a section without tumor present, the tumor tissue and the cerebellum region is still identified as being different from all tissue within the training data (Figure S1). This means that this approach could be used to distinguish outlier from training data, serve as a quality control metric, or be used to identify data of interest for additional analysis.

The consistent embedding provided by our approach can also be applied to nonimaging mass spectrometric data. A promising new area of mass spectrometric research is rapid evaporative ionization mass spectrometry (REIMS).^35^ This can classify tissues during surgeries in almost real time. In addition to this, it can be used for rapid cell profiling.^36^ Neural network learned t-SNE was then performed on REIMS data acquired from cell pellets extracted from the small intestine and colon of mice with different oncogenetically relevant mutations. There is a much clearer separation in the genetic types observed by this method as compared to PCA (Figure S2). These data were then used as a basis for classification by linear discriminant analysis (LDA). The classification based on the NN t-SNE reduced data shows much higher accuracies as compared to data reduced by PCA (Rand index 0.93 compared to 0.83, Table S2), indicating that this might be a more powerful dimensionality reduction method to apply prior to classification. It is important to note that a normal t-SNE approach would be inadequate for classification because new data cannot be projected into the reduced space. The clinical application of this approach is clear—it allows real-time stratification of patients/mice into groups based upon multidimensional data sets, rather than binary segregation based upon presence or absence of individual dominant oncogenic mutations.

#### Large Data t-SNE

The challenge of applying nonlinear dimensionality reduction to larger data sets that cannot be loaded into RAM is also met by this approach, broadening the applicability of this method. This has been addressed previously by hierarchical t-SNE and LION-tSNE described previously.^12,13^ Using the our approach is another way to achieve this with greater efficiency than these existing methods. We demonstrated this using a publicly available chromosome conformation capture data set containing over 2 million samples.^37^ This method is able to rapidly produce informative results on these large data that could not be analyzed using standard t-SNE methods (Figures 4 and S3). Hi-C is a DNA sequencing-based method for probing the 3D structure and interactions of chromosomes. This type of data is notoriously challenging to load into RAM and process, even on modern computers. We performed a three-dimensional embedding of the entire Hi-C data set for a human GM12878 cell line^37^ using the highest possible resolution of 1 kb. The resulting embedding captured the spatial relationships between the chromosomes encoded in the Hi-C data set (Figure 4a). Not surprisingly, the 22 chromosomes exhibit more intrachromosomal interactions than interchromosomal interactions. Moreover, this approach more accurately captured the hierarchical nature of the human nucleus, which is divided into discrete A/B subcompartments, described previously.^37^ These subcompartments are regions of chromosomes which harbor different epigenetic marks and display a tendency to physically interact with regions in the same subcompartment type. After annotating our 3D embedding with the H3K27ac ChIP-seq signal (H3K27ac is an epigenetic mark linked to active gene expression), we observed that regions of high transcriptional activity cocluster together in the 3D space (Figure S3). This highlights the hierarchy and complexity encoded in our unsupervised embedding and suggests that using this approach can further aid studies of the human genome architecture, for example in identifying novel genome subcompartment types which could be missed using lower resolution data. In order to perform t-SNE on these data, over 80 TB of RAM would be required, making the analyses on these types of data sets infeasible. The subsampling by the our approach meant that less than 10 GB are required at any one time to segment these data.

**Figure 4:**
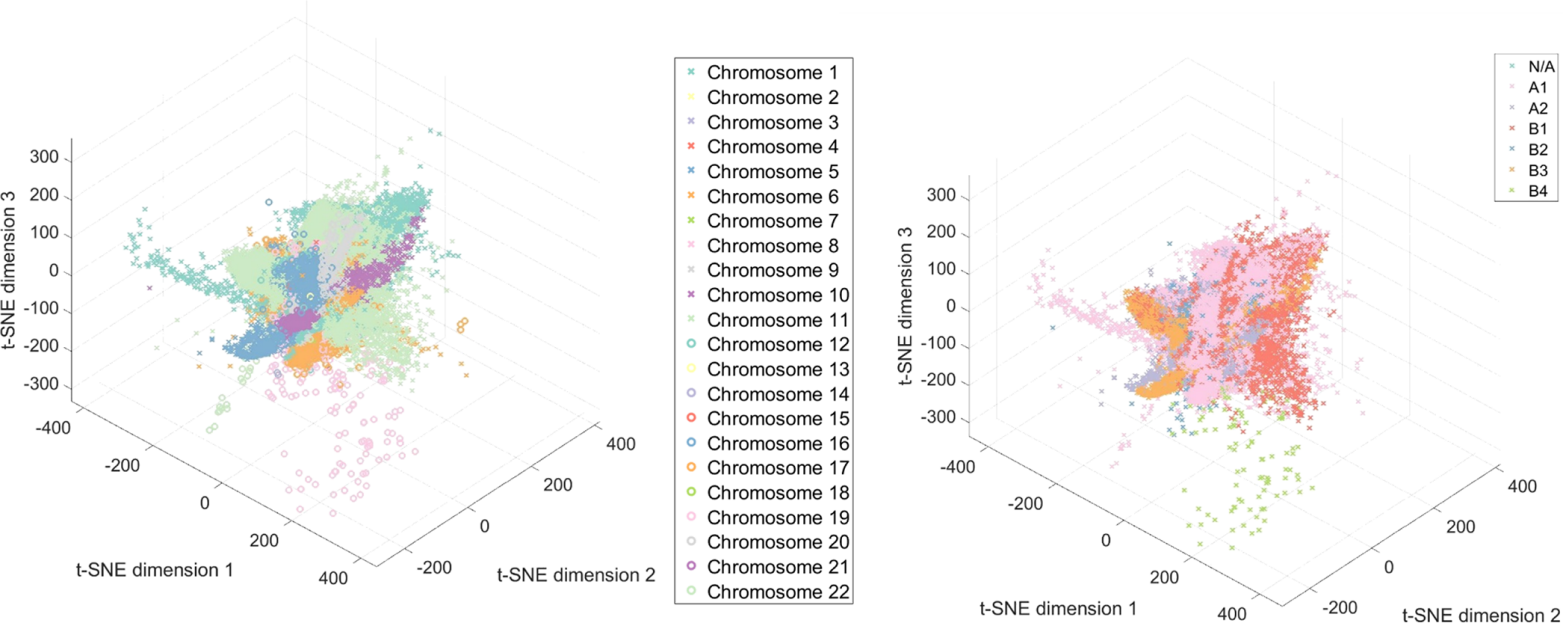
Results of 3D embedding from NN t-SNE dimensionality reduction on Hi-C data containing over 2 million samples labeled by chromosome (A), or interactions (B). Using standard t-SNE approaches would not be possible for either this data set due to memory constraints, and simply loading these data into RAM is often not possible.

#### Combining These Attributes (Large Data, Consistent Segmentation, and Spectral Information)

When acquiring data from biological samples, technical and biological replicates are important. Even just performing these in duplicate, this means that for any single tissue type, four tissues may be analyzed. Adding multiple genetic variants and tissue types further increases the amount of data for comparison. This was demonstrated on a MALDI data set from the colon of four genetic variants of genetically engineered mouse models (wild-type, Apc deficient, Kras^G12D^ mutant, and dual Apc deficient with Kras^G12D^ mutation), analyzed with duplicate biological and technical replicates (total of 24 tissue sections). This data set contained 427,967 pixels and 4,000 spectral features. Using the NN learned t-SNE, we see that there is a clear cluster relating to the KRAS mutation containing mice (pink cluster Figure 5), a cluster which is primarily present in the mice with Apc deficient intestinal tissue (dark blue cluster Figure 5) and a cluster that relates to all but the wild type mice (light blue cluster Figure 5). The main drivers of these differences were *m*/*z* 885.550 (light blue), 514.280 (dark blue), and 835.530, 861.550, and 887.566 (pink). These masses were then matched to the HMDB within 5 ppm mass error to provide tentative assignments of PI (38:4) [M-H]^−^ (*m*/z 885.550) taurocholic acid [M-H]^−^ (*m*/z 514.280), and PI (34:1), (36:2), and (38:3) [M-H]^−^ respectively (*m*/z 835.530, 861.550, 887.566). The PI species identified in this study have clear biological relevance, with PI (38:4) known to be the most abundant mammalian phosphatidyl inositol.^38^ Phosphatidyl inositide species are critical cell signaling secondary messenger molecules whose relative abundance is both influenced by oncogene or tumor suppressor mutations (such as PIK3CA or KRAS mutations or PTEN loss), and are critical for characteristic tumor cell phenotypes including unrestrained growth and survival signaling. Similarly, the identification of the purple cluster associated with Apc deficiency driven by a secondary bile acid such as (tentatively assigned) taurocholic acid may give valuable insight into the comparative biology of transformed versus normal intestinal enterocytes and relate to the critical role played by the gut microbiome in initiation and progression of colorectal cancer.^39^

**Figure 5:**
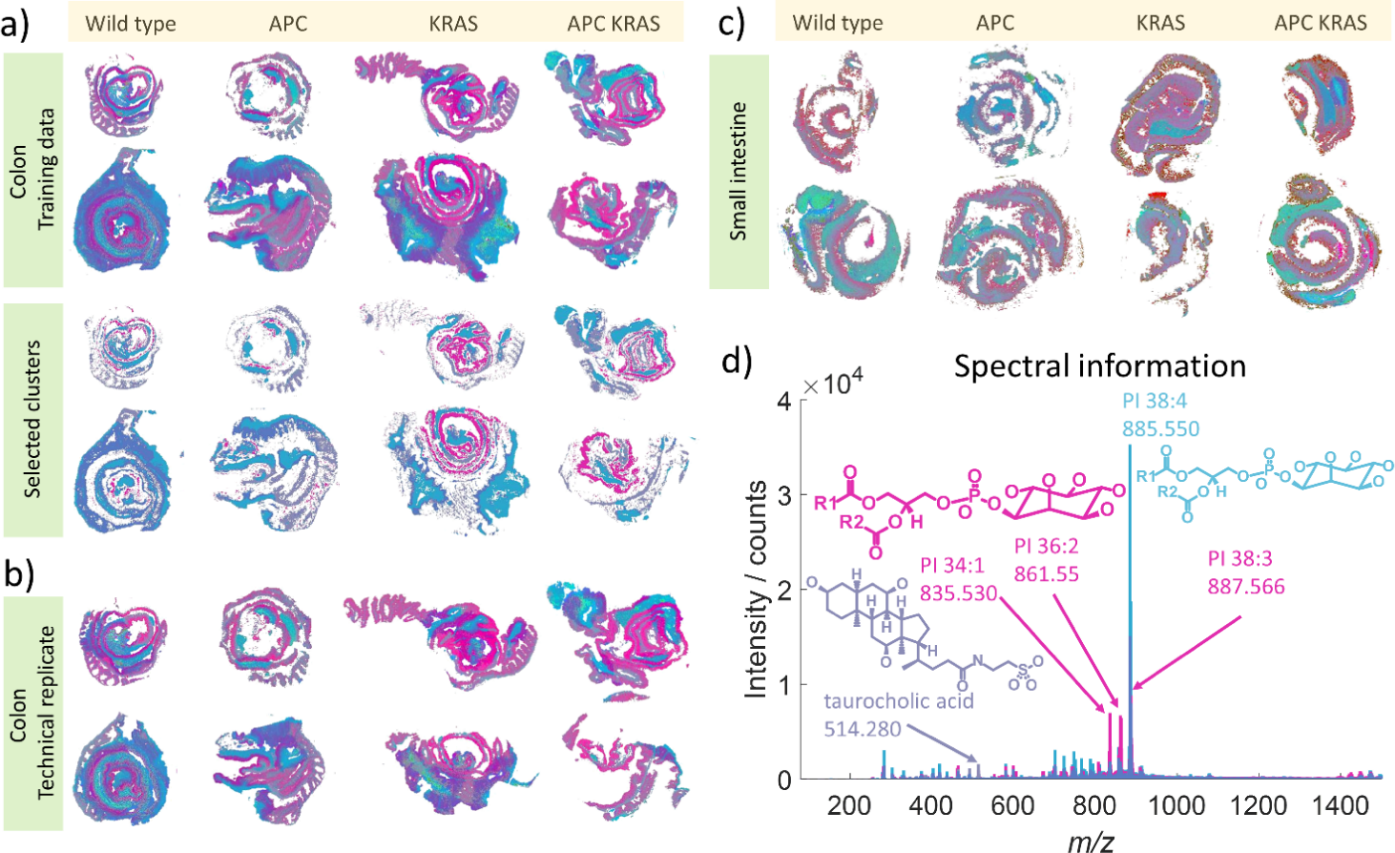
Results of neural network t-SNE applied to MSI data from biological and technical replicates from a genetically engineered mouse models for colorectal cancer. The RGB color space from the NN t-SNE clearly differentiates the genetic differences based on difference in the metabolites detected by MSI. The pink cluster is observed primarily in the KRAS mutation containing tissues, while the purple cluster is prevalent in the APC deletion containing tissues. These clusters are driven by phosphatidyl inositol lipids and taurocholic acid, respectively. This segmentation is consistent in technical replicated (b), and additional data from small intestine can be compared with comparable color segmentation (c).

#### Multiomics Data Integration

As well as being capable of learning the transformation within a single data type, there is no limitation that the input data for training the neural network must be of the same type as the reduced data. Therefore, with matched samples, we can use the same methodology to learn embedding between different types of omics. To demonstrate this we use data from metabolomics (REIMS) and transcriptomics of cell pellets from breast cancer associated cell lines with different estrogen, progesterone, and human epidermal growth factor receptor status.^40^ By applying t-SNE to the metabolic data, the different cell lines can be stratified (Figure 6a) but do not cluster according to the associated receptor status (Figure 6b). In comparison, t-SNE applied to the transcriptomics data clearly differentiates the ER receptor status (Figure 6c). By training neural networks to integrate the metabolic data into the t-SNE space of the transcriptomics data, the estrogen receptor (ER) status can now be differentiated from the metabolic data (Figure 6d). This can be used as the basis for classification on the metabolic data. To test this, we used leave one out classification testing on the metabolic data with different reduction methods. The neural network integration improves the receptor status classification accuracy of the metabolic data for ER and triple negative (TN) status by 15% and 10%, respectively (Table S3). This could be further extended by inclusion of other omics such as genomics that might also differentiate the human epidermal growth factor receptor 2 (HER2) and progesterone receptor (PR) status. As described previously, we can also identify the metabolic drivers of these differences associated with receptor status. The driving ions of ER and TN status are summarized in Tables S4 and S5. In short, the primary drivers detected in REIMS are lipids and fatty acids corroborating those described previously.^40^ In comparison the use of PCA scores to determine molecular drivers of differences does not identify the fatty acids determined using the our method (Table S6). Using the NN t-SNE to just reduce the REIMS data alone (Figure 6a) and returning spectral drivers of the different receptor statuses identifies similar molecules to using PCA on the REIMS data (Tables S7 and S8). This highlights the benefit of being able to project the REIMS data into the transcriptomics reduced space enabling not only better classification accuracy but also a more informative review of the ions driving differentiation.

**Figure 6:**
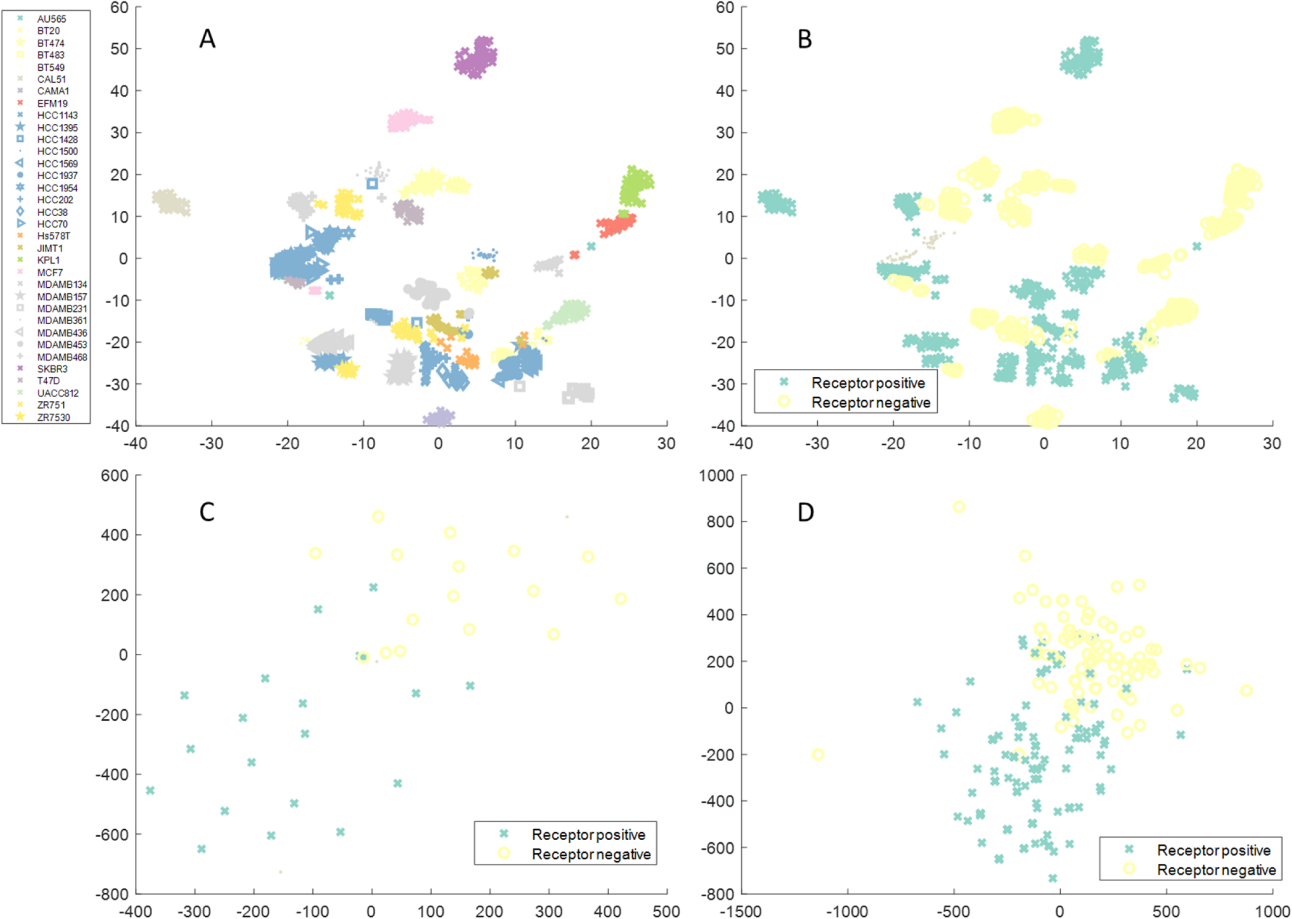
Comparison of reduction of metabolic (REIMS) and transcriptomic data using t-SNE. The cell lines can be well separated according to their metabolic profiles (A), but do not differentiate ER status (B). In comparison, t-SNE applied to the transcriptomics data differentiates the data according to the ER status (C). By using neural networks, the metabolic data can be reduced to the transcriptomics embedding space, now differentiating the ER status.

### Extension to Other Dimensionality Reduction Methods

Finally, the new approach is not limited to using t-SNE as the basis for dimensionality reduction. Since the UMAP algorithm has shown recent popularity in many different areas we demonstrate the use of neural network learning of UMAP embedding on the vast chromosome conformation capture data shown in Figure 4. As with t-SNE, this deep learning training approach is able to reduce the Hi-C data into 3D showing the same segmentation based primarily intrachromosome interaction (Figure S4a), with additional hierarchical interactions, and transcriptional activity clustering (Figure S4b,c). This approach is generalizable to different dimensionality reduction and demonstrates training of deep learning on nonlinear dimensionality reduction. Since the field of nonlinear dimensionality reduction is continually growing, this approach is applicable to other future developments in this area. This is also applicable to the use of neural network learning to integrate data from multiple omics data. To demonstrate this, we have applied UMAP to the transcriptomics data described previously and trained neural networks to learn the embedding from the REIMS data to this reduced space (Figure S5). As with the use of t-SNE, the REIMS data separates according to receptor status when reduced to the corresponding transcriptomics space (Figure S5d), and an improvement in classification accuracy via LDA can be achieved compared to LDA performed on the UMAP directly on the REIMS data (Table S9).

## Conclusions

The method described here overcomes the major limitations of conventional nonlinear reduction methods. The proposed method can be performed on large data sets, and return consistent embedding with the ability to incorporate new data into the embedded space, and return the driving spectral contributors to the changes observed. This method can also be used to perform multiomics data integration by training one omics data set to the reduced space of another. This presents a new way to more effectively mine and analyze high dimensional omics and multiomics data that has large sample numbers. This principle has application across significant scientific domains, and we have exemplified its use here by deploying it on data sets from different fields including Hi-C, mass spectrometry imaging, and transcriptomics. We selected these examples as they could not be handled by t-SNE or UMAP. Through several case studies we show how this method successfully extends the use of t-SNE and UMAP to data sets containing previously prohibitively large numbers of samples and provides a means to derive additional understanding from these large data sets. Through several case studies we show how this method successfully extends the use of t-SNE and UMAP to data sets containing previously prohibitively large numbers of samples and provides a means to derive additional understanding from these large data sets. We show how additional data can be added, posthoc. In many of the fields where nonlinear reduction is currently applied, such as genomics and proteomics, data is expected to soon exceed the current capacity of existing algorithms. For example, the UK Biobank currently contains genomics data from 487,401 samples, which is far greater than conventional methods can analyze.^41^ These large-scale initiatives will continue to acquire large cohort data sets from many different techniques, and algorithms need to develop to allow processing and mining of these data. Future considerations for this work include additional comparison of subset sampling methods (such as Sobol sampling) and applications to other areas of research such as large-scale genomics initiatives. We have benchmarked the performance of this approach against using standard t-SNE using the correlation metric between the original t-SNE and NN trained t-SNE with *r* ^2^ > 0.95 between the two approaches both for the forward and reverse transformations.

## Supplementary Material



## Data Availability

All code relating to this publication can be found at https://github.com/NiCE-MSI/Neural-network-dimensionality-reduction.

## Notes

Method parameter optimization including subset size, hyperparamater selection for the initial reduction, subsampling approach, and the neural network training method; tables of the classification evaluation on the REIMS and transcriptomics data, and associated molecular drivers; additional figures of the embeddings using UMAP rather than t-SNE as the initial dimensionality reduction approach (PDF)
