# Supplementary material for: A New Approach to Large Multiomics Data Integration
Source: Anal Chem. 2025 Sep 11;97(37):20058–67. doi: 10.1021/acs.analchem.5c01812 (PMC12461686; doi:10.1021/acs.analchem.5c01812)
Supplement: Supplementary file 1 [file ac5c01812_si_001.pdf]

Supplementary Materials for

A new approach to large multi-omics data integration

Authors: Alex Dexter[1], Spencer A. Thomas[1], Rory T. Steven[1], Kenneth N. Robinson[2], Adam J. Taylor[3], Efstathios A. Elia[4], Chelsea Nikula[1], Andrew D. Campbell[5], Yulia Panina[6], Arafath K. Najumudeen[7], Bin Yan[1], Piotr Grabowski[8], Gregory Hamm[9], John Swales[9], Aurelien Tripp[10], George Poulogiannis[10], Mariia O. Yuneva[6], Simon Barry[9], Richard J.A. Goodwin[9], Owen J. Sansom[5], Zoltan Takats[11], and Josephine Bunch[1,11] *

Affiliations:

[1] National Physical Laboratory, Teddington, UK, TW11 0LW

[2] Bactobio, London, UK, SE11 5JH

[3] Sage Bionetworks, Seattle, WA, USA, 98121-1031

[4] Department of Chemistry, University of Cyprus, 2109, Nicosia, Cyprus.

[5] Beatson Cancer Research UK Institute, Glasgow, UK, G12 0YN

[6] The Francis Crick Institute, London, UK, NW1 1AT

[7] Institute for Molecular Medicine Finland (FIMM), HiLIFE, University of Helsinki, Helsinki, Finland, 00290

[8] Biological Insights Knowledge Graph, AI Strategy and Innovation, R&D IT, AstraZeneca, Barcelona, Spain, 08028

[9] Integrated Bioanalysis, Clinical Pharmacology & Safety Sciences, R&D, AstraZeneca, Cambridge, UK, CB2 0AA,

[10] The Institute of Cancer Research, London UK, SW3 6JB

[11] Department of Metabolism, Digestion and Reproduction, Imperial College London, London, UK, W12 0NN

*Correspondence to: josephine.bunch@npl.co.uk

This file includes:

Description of parameter optimization for the method
Figures. S1 to S15
Tables S1 to S9

Parameter optimisation

There are several considerations to be taken into account when applying the our method to different datasets. One of these is the effect of the size of the subset on the accuracy of the segmentation. Due to the stochastic nature of t-SNE, performing it on different subsets will give different results each time, making evaluation of the segmentation challenging. Therefore, a smaller dataset was used, on which t-SNE was performed using the whole data. Subsets of the final reduced data were then taken as the input for training a neural network. The result of the neural network trained t-SNE was then compared to the t-SNE on the complete dataset by means of the correlation of the resulting reduced three-dimensional space. This was performed by subsampling the data both randomly and in an ordered manner.

The effect of random or ordered subsampling seems to have little to no effect on the end result in this instance (Figure S6 and S7), as both methods converge to give the same correlation to the original t-SNE (within error bars) from around 10% subset size, and similar anatomical segmentation. Efficient subsampling to give subsets that are representative of the whole data would be an interesting topic for a future study. For example, in image analysis, Sobol series sampling has been used to minimise the unsampled areas of the image when performing a subsampling for PCA(*51*). We note however upon further examination of the data presented in Figure 5, that the subsampled t-SNE space occupies a similar space to the final embedding (Figure S8), which indicates that the subsampling in this instance has effectively captured the full range of data within the whole data. To ensure consistency of these results we have also tested the correlation between the original t-SNE and NN learned embedding when we used alternative hyperparamater selection for t-SNE (perplexity of 1000, and exaggeration of 1), and found the correlation between then to remain above 0.95 (0.9577 for t-SNE and 0. 9925 for UMAP). Furthermore, the anatomical segmentation remains clear for these parameters (Figure S9).

To analyse the effect of subset size on the whole algorithm, including the t-SNE portion, well known samples are required. Towards this end, sagittal, transverse, and coronal mouse brain tissues were chosen as they have very well-defined anatomical features. These were acquired with a variety of different pixel sizes (100, 45 and 20 μm) to give a total number or pixels ranging from 10,000 to 100,000. NN t-SNE was then applied to subsets of these datasets ranging from the whole down to 0.1% of the data (Figure S10). As might be expected, the larger the subset of data, the better the neural network training. The performance is not linear however, and below a subset size of 1,000 pixels the segmentation performs very poorly compared to the expected anatomical features (Figure S10). The measure of image autocorrelation introduced for MSI by Smets *et al.(52)* was also used to evaluate the results from the subset size comparison on the sagittal brain dataset, and similarly we see a large decrease in the correlation between subsets of 600-200 pixels (0.5% and 0.2%, Figure S11). This is independent of the size of the original data, making this method particularly well suited for datasets with large sample numbers (pixels).

The analysis of these images shows that with larger datasets, smaller subsets can be used, and the same quality of embedding can be achieved. The sagittal brain dataset with 123,557 pixels shows similar embedding with 618 pixels in the subset (0.5% subsampling) and 12,356 pixels (10%

subsampling) whereas the quality of the segmentation in the coronal and transverse brain images declines rapidly below 1478 and 930 pixels (5 and 10%), respectively. Since the performance is more dependent on the absolute subset size rather than the percentage, a subset of at least 2,000 pixels is recommended. Notably, larger subsets are not required for the data containing 1,000,000 pixels compared to 10,000. It is also worth noting that the time taken to perform t-SNE increases quadratically with increasing number of pixels whereas the neural network training is linear, and generally requires much less time (figure S12). Therefore, the rate limiting step in this method remains the application of t-SNE to the subset of data. A similar trend can be observed in the RAM usage of the different steps (figure S13). Where the t-SNE step is also the major limiting factor in the processing larger subsets of data.

The other consideration is how accurately the reconstruction of a spectrum from the t-SNE space can be achieved. In order to evaluate the success of these results single pixel spectra were compared to the returned spectra from their respective RGB points using the NN t-SNE method (Figure S14). This was demonstrated on six spectra that were randomly selected from the colorectal cancer dataset in Figure 5. In all cases a very high correlation ($r^2 > 0.9$) between the original and the returned spectra were observed.

The final consideration for this approach is the neural network training parameters. There are a large number of possible combinations that could be altered to varying effect. To exhaustively study this landscape is beyond the scope of this investigation, however a preliminary evaluation of the different neural network training algorithms was carried out. Twelve different algorithms were investigated, and the correlation between original t-SNE and NN trained t-SNE was used to evaluate this (Figure S15), demonstrating that Bayesian regularisation and Levenberg-Marquardt outperform all other algorithms ($r^2 > 0.9$ compared to < 0.75 for all other methods) with Bayesian regularisation having slightly better performance of the two. This is likely because this method is robust towards overfitting which can occur due to the high dimensionality of MSI and other data(*53*).

Table S1. Summary of experimental parameters for the acquisition of the mass spectrometry imaging datasets.

**Dataset**	**Ionisation source**	**Pixel size / µm**	***m/z* Range**	**Matrix/solvent**	**Scan speed / pixels per second**	**Polarity**
Breast cancer model	DESI	75	50-1500	95/5 MeOH/water 0.02 mg/ml raffinose	2	Negative
Sagittal brain	MALDI	20	100-1500	CHCA	20	Positive
Transverse brain	DESI	50	50-1200	95/5 MeOH/water	4	Positive
Coronal brain	MALDI	45	100-1200	CHCA	2	Positive
Colorectal cancer model	MALDI	50	50-1500	9-AA	2.5	Negative
Glioblastoma	MALDI	50	100-1000	2,5-DHB	25	Positive

Figure S1. Results of NN-tSNE trained on the 21st section (highlighted in red) of a 3D brain dataset which does not contain glioblastoma (which is later segmented in dark blue) and subsequently applied to the data from the remaining sections showing a consistent segmentation of the similar anatomy. More importantly the pixels from the glioblastoma are identified as being different from any data used in the training process. Sections are ordered by depth left to right and bottom to top, and the approximate corresponding image from the Allen brain atlas is sown below each image.

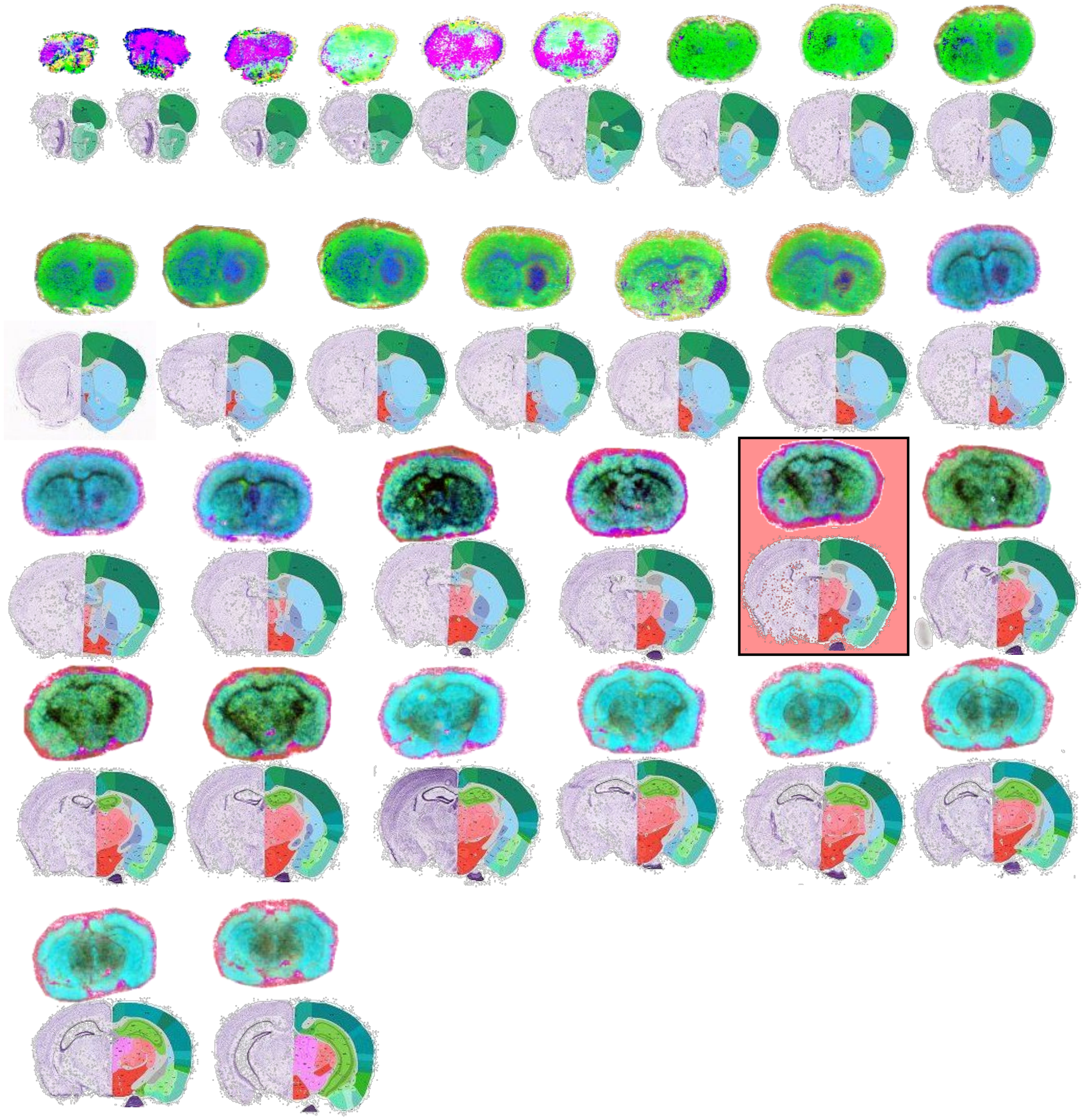

Figure S2. Comparison of NN-tSNE and PCA applied to REIMS data from cell pellets collected from colon (x) and small intestine (o) from genetic mouse models. An additional nine blinded cell pellets were also collected (black) which were used to evaluate the classification using these approaches. NN t-SNE shows much clearer segmentation of the different genotypes based on their metabolic profiles than PCA.

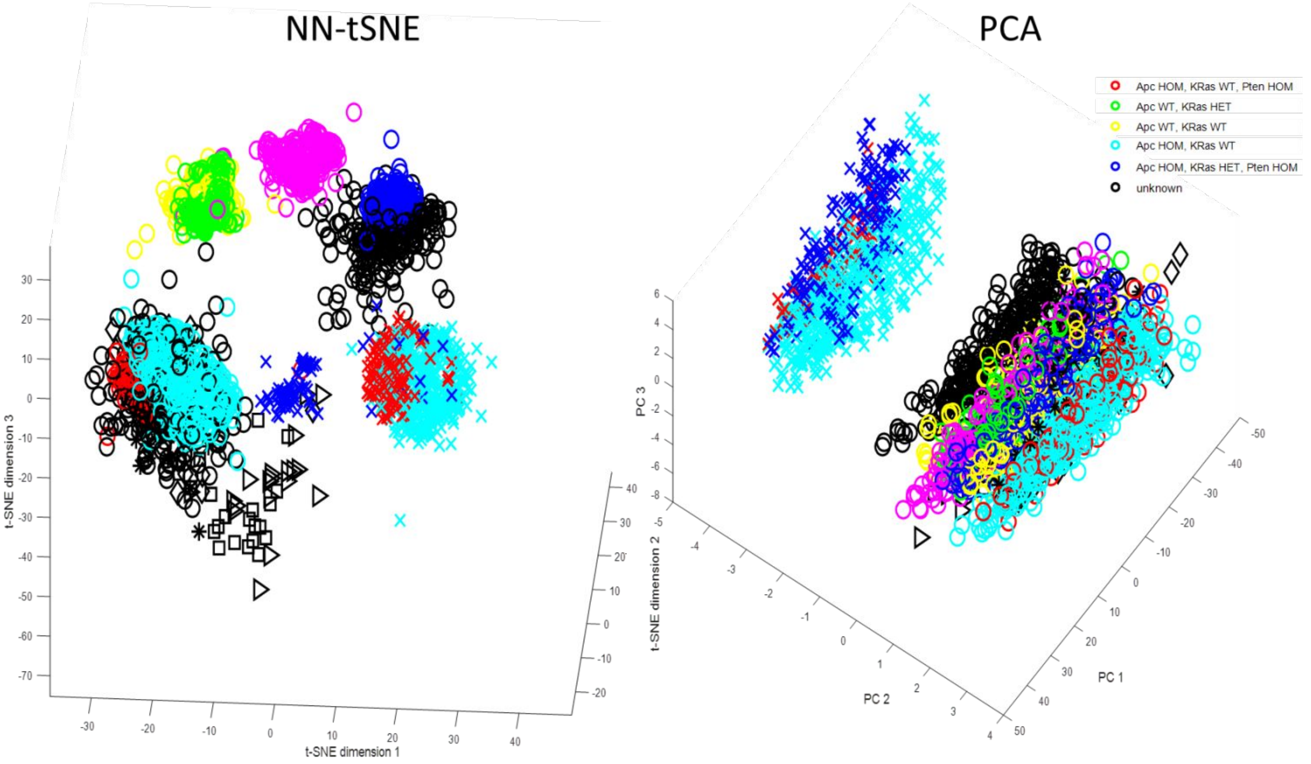

5

Table S2. Comparison of classification results using either PCA or NN-tSNE as dimensionality reduction prior to LDA classification training. Classification on the NN-tSNE reduced data outperforms the PCA reduced in all metrics on these data.

		'Apc HOM, KRas HET'	'Apc HOM, KRas HET, Pten HOM'	'Apc HOM, KRas WT'	'Apc HOM, KRas WT, Pten HOM'	'Apc WT, KRas HET'	'Apc WT, KRas WT'	Average
PCA/LDA	Rand index	0.852823731	0.777524244	0.719908728	0.882487165	0.903593839	0.840273816	0.829435
	Jaccard index	0.289256198	0.362745098	0.48042328	0	0.150753769	0.138461538	0.23694
	True positive rate	0.446808511	0.513888889	0.813620072	0	0.20979021	0.251396648	0.372584
NN-tSNE/LDA	Rand index	0.963491158	0.954934398	0.86081004	0.899600685	0.962920707	0.93953223	0.930215
	Jaccard index	0.764705882	0.818390805	0.678947368	0.238095238	0.636871508	0.563786008	0.616799
	True positive rate	0.885106383	0.824074074	0.924731183	0.266990291	0.797202797	0.765363128	0.743911

Figure S3. Results of NN t-SNE on Hi-C data labelled by the H3K27ac ChIP-seq signal (epigenetic mark linked to active gene expression). Regions with high transcriptional activity (yellow) are clustered together in the 3D space.

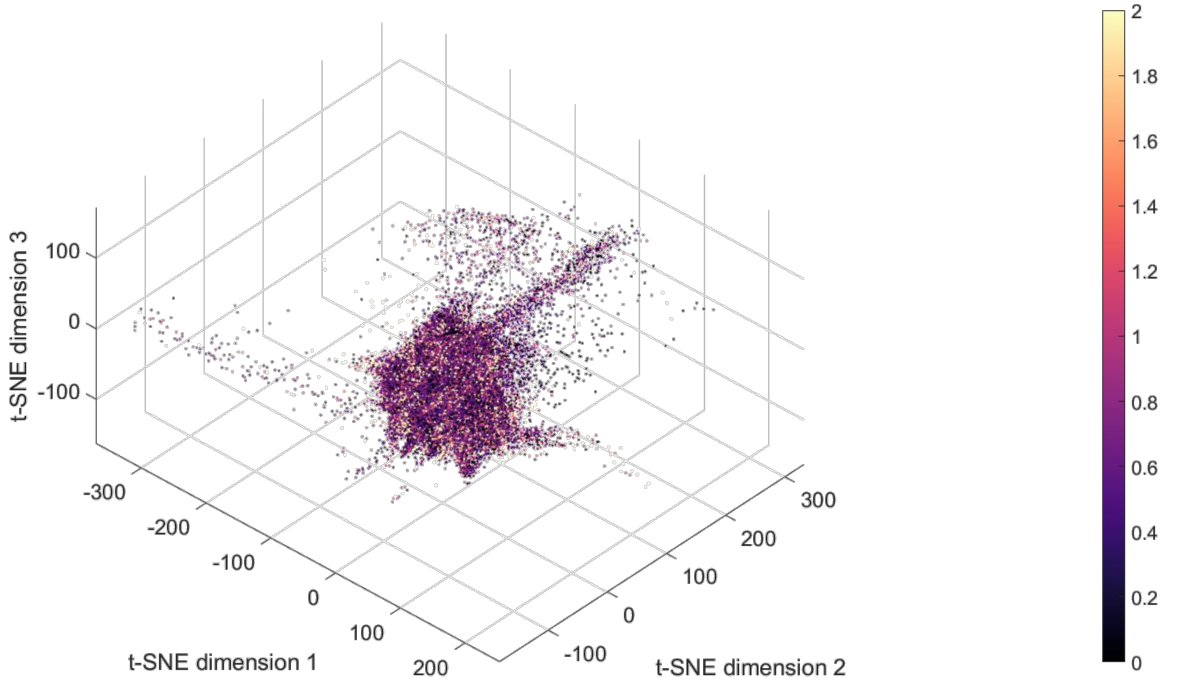

Table S3. Leave one out classification errors (%) for linear discriminant analysis (LDA) and quadratic discriminant analysis (QDA) on the t-SNE reduction of the REIMS data alone, PCA on the REIMS data, and the neural network projected REIMS data into transcriptomics t-SNE space. The REIMS data projected into transcriptomics t-SNE has lower errors for the ER and TN status by ~20 and 10% respectively.

Classification method	ER error %	HER2 error %	PR error %	TN error %
LDA on REIMS t-SNE	33	34	28	25
QDA on REIMS t-SNE	30	28	22	16
LDA on REIMS PCA	35	33	22	32
QDA on REIMS PCA	45	34	23	52
LDA on REIMS projected into transcriptomics	15	34	23	9
QDA on REIMS projected into transcriptomics	13	34	23	10

Table S4. Summary of the top 10 ions that are associated with increased and decreased regulation in the ER positive status as determined using the returned spectral information using our method to embed the REIMS data into the transcriptomics t-SNE space. Tentative assignments are made to the HMDB with ppm error to the exact masses shown.

**Downregulated in ER positive**				**Upregulated in ER positive**			
m/z	Assignment	Adduct	Ppm error	*m/z*	Assignment	Adduct	Ppm error
671.46	PA(34:2)	[M-H]$^-$	3	699.50	PA(36:2)	[M-H]-	3
645.45	PA(32:1)	[M-H]$^-$	3	223.03	N-Acetylglutamine	[M+Cl]-	90
673.48	PA(34:1)	[M-H]$^-$	4	744.55	PE(36:1)	[M-H]-	3
716.52	PE(34:1)	[M-H]$^-$	4	863.56	PI(36:1)	[M-H]-	3
742.54	PE(36:2)	[M-H]$^-$	3	727.53	PA(38:2)	[M-H]-	3
672.47	PC(28:2)	[M-H]$^-$	9	700.50	PC(30:2)	[M-H]-	8
325.18	Dihydrotestosterone	[M+Cl]$^-$	34	735.47	PA(36:2)	[M-H]-	4
253.22	Palmitoleic acid	[M-H]$^-$	5	165.04	Unknown	N/A	N/A
572.48	Cer(d34:1)	[M-H]$^-$	4	193.05	Unknown	N/A	N/A
718.54	PE(34:0)	[M-H]$^-$	3	766.54	PE(34:4)	[M-H]-	3

Table S5. Summary of the top 10 ions that are associated with increased and decreased regulation in the TN receptor status as determined using the returned spectral information using our method to embed the REIMS data into the transcriptomics t-SNE space. Tentative assignments are made to the HMDB with ppm error to the exact masses shown.

**Downregulated in TN**				**Upregulated in TN**			
m/z	Assignment	Adduct	Ppm error	*m/z*	Assignment	Adduct	Ppm error
699.50	PA(36:2)	[M-H]-	3	671.46	PA(34:2)	$[M-H]^-$	3
744.55	PE(36:1)	[M-H]-	3	673.48	PA(34:1)	$[M-H]^-$	4
727.53	PA(38:2)	[M-H]-	3	645.45	PA(32:1)	$[M-H]^-$	3
642.49	LysoPC(28:1)	$[M-H_3O]^-$	2	659.46	PA(33:1)/ DG(40:10)	$[M-H]^-$	3/8
745.56	PG(34:2)	[M-H]-	72	672.47	PC(28:2)	$[M-H]^-$	9
223.03	N-Acetylglutamine?	[M+Cl]-	90	835.53	PI(34:1)	$[M-H]^-$	4
772.58	PE(38:1)	[M-H]-	3	861.55	PI(36:2)	$[M-H]^-$	2
700.50	PC(30:2)	[M-H]-	8	281.25	Oleic acid	$[M-H]^-$	6
717.51	PA(38:7)	[M-H]-	77	674.48	PC(28:1)	$[M-H]^-$	9
766.54	PE(34:4)	[M-H]-	3	685.48	PA(35:2)/ DG(42:11)	$[M-H]^-$	3/7

Table S6. Summary of the top 10 ions that are increased or decreased in principal component 2 of the REIMS data that shows some but not complete separation of the TN receptor status (Figure 6). Tentative assignments are made to the HMDB with ppm error to the exact masses shown.

**Increased in PC 2**				**Decreased in PC 2**			
m/z	Assignment	Adduct	Ppm error	*m/z*	Assignment	Adduct	Ppm error
643.432	PA(32:2)	$[M-H]^-$	4	699.50	PA(36:2)	[M-H]-	3
223.03	N-Acetylglutamine	[M+Cl]-	90	700.50	PE-NMe(32:2)	[M-H]-	8
645.45	PA(32:1)	$[M-H]^-$	3	727.53	PA(38:2)	[M-H]-	3
689.47	PA(P-34:1)	$[M-H]^-$	3	735.47	PA(36:2)	[M+Cl]-	4
717.52	DG(40:10)	$[M+Cl]^-$	10	725.51	PA(38:3)	[M-H]-	2
672.47	PC(28:2)	$[M-H]^-$	9	673.48	PA(34:1)	$[M-H]^-$	4
697.48	PA(36:1-O)	$[M-H]^-$	1	728.53	PC(32:2)	[M-H]-	9
742.54	PE(36:2)	$[M-H]^-$	3	701.51	PA(36:1)	[M-H]-	4
716.52	PE(34:1)	$[M-H]^-$	4	772.58	PE(38:1)	[M-H]-	3
671.46	PA(34:2)	$[M-H]^-$	3	687.50	DG(42:10)	[M-H]-	6

Table S7. Summary of the top 10 ions that are associated with increased and decreased regulation in the ER status as determined using the returned spectral information using our method to embed the REIMS into t-SNE space (not from the transcriptomics). Tentative assignments are made to the HMDB with ppm error to the exact masses shown.

**Downregulated in ER positive**				**Upregulated in ER positive**			
m/z	Assignment	Adduct	Ppm error	*m/z*	Assignment	Adduct	Ppm error
699.50	PA(36:2)	[M-H]-	3	743.54	PA(38:1-O)	[M-H]$^-$	23
673.48	PA(34:1)	[M-H]$^-$	4	846.66	GlcCer(d42:1)	[M-H]$^-$	2
700.50	PC(30:2)	[M-H]-	8	766.54	PE(34:4)	[M-H]-	3
687.50	DG(42:10)	[M-H]-	6	863.56	PI(36:1)	[M-H]$^-$	3
717.51	PA(38:7)?	[M-H]-	77	772.58	PE(38:1)	[M-H]-	3
735.47	PA(36:2)	[M+Cl]-	4	717.52	DG(40:10)	[M+Cl]$^-$	10
674.48	PC(28:1)	[M-H]$^-$	9	669.449	PA(34:3)	[M-H]$^-$	2
701.51	PA(36:1)	[M-H]-	4	643.432	PA(32:2)	[M-H]$^-$	4
744.55	PE(36:1)	[M-H]-	3	742.537	PE(36:2)	[M-H]$^-$	3
713.51	DG(38:3-2OH)	[M+Cl]-	4	716.52	PE(34:1)	[M-H]$^-$	4

Table S8. Summary of the top 10 ions that are associated with increased and decreased regulation in the TN receptor status as determined using the returned spectral information using the our method to embed the REIMS into t-SNE space (not from the transcriptomics). Tentative assignments are made to the HMDB with ppm error to the exact masses shown.

**Downregulated in TN positive**				**Upregulated in TN positive**			
m/z	Assignment	Adduct	Ppm error	*m/z*	Assignment	Adduct	Ppm error
716.52	PE(34:1)	$[M-H]^{-}$	4	659.46	PA(33:1)/ DG(40:10)	$[M-H]^{-}$	3/8
742.537	PE(36:2)	$[M-H]^{-}$	3	713.51	DG(38:3-2OH)	[M+Cl]-	4
643.432	PA(32:2)	$[M-H]^{-}$	4	701.51	PA(36:1)	[M-H]-	4
669.449	PA(34:3)	$[M-H]^{-}$	2	674.48	PC(28:1)	$[M-H]^{-}$	9
863.563	PI(36:1)	$[M-H]^{-}$	3	735.47	PA(36:2)	[M+Cl]-	4
772.58	PE(38:1)	[M-H]-	3	717.51	PA(38:7)?	[M-H]-	77
717.52	DG(40:10)	$[M+Cl]^{-}$	10	687.50	DG(42:10)	[M-H]-	6
766.54	PE(34:4)	[M-H]-	3	700.50	PC(30:2)	[M-H]-	8
743.54	PA(38:1-O)	$[M-H]^{-}$	23	673.48	PA(34:1)	$[M-H]^{-}$	4
846.658	GlcCer(d42:1)	$[M-H]^{-}$	2	699.50	PA(36:2)	[M-H]-	3

5

Table S9. Leave one out classification errors (%) for linear discriminant analysis (LDA) and quadratic discriminant analysis (QDA) on the t-UMAP reduction of the REIMS data alone, PCA on the REIMS data, and the neural network projected REIMS data into transcriptomics UMAP space. The REIMS data projected into transcriptomics UMAP has lower errors for the ER and TN status by ~15 and 25% respectively.

**Classification method**	**ER error %**	**HER2 error %**	**PR error %**	**TN error %**
LDA on REIMS UMAP	30	27	24	35
QDA on REIMS UMAP	29	36	20	41
LDA on REIMS PCA	35	33	22	32
QDA on REIMS PCA	45	34	23	52
LDA on REIMS projected into transcriptomics	17	34	19	10
QDA on REIMS projected into transcriptomics	17	34	17	10

Figure S4. Results of neural network training on UMAP applied to a subset of the Hi-C data seen in figure 4. As with the neural network t-SNE the NN-UMAP primarily segments the data based on chromosome number (a), with other detail being discerned such as the A/B sub-compartments (b) and co-clustering transcriptional activity (c).

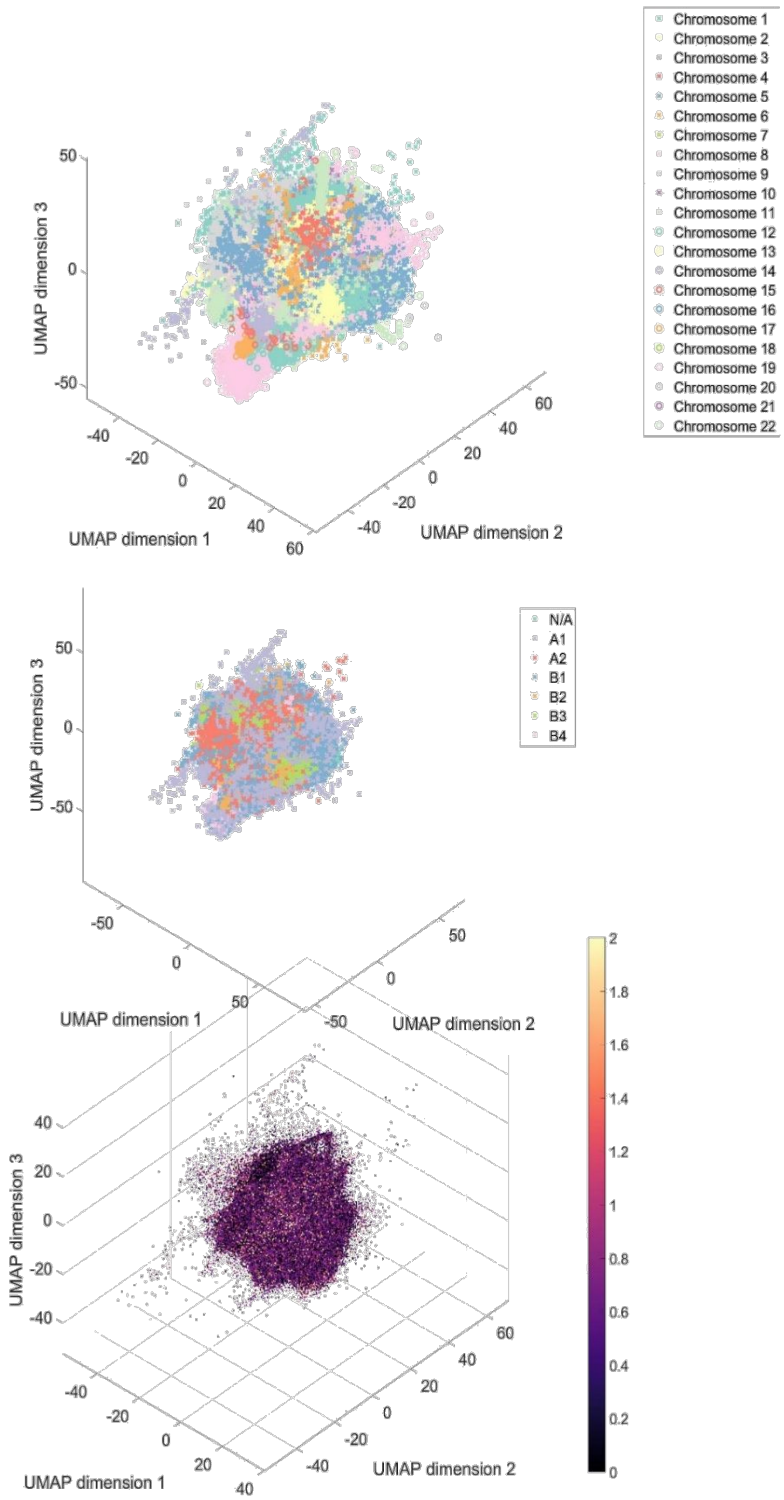

Figure S5. Comparison of reduction of metabolic (REIMS) and transcriptomic data using UMAP. Unlike with t-SNE, the cell lines cannot be well separated according to their metabolic profiles (A), and still do not differentiate ER status (B). As with t-SNE, UMAP applied to the transcriptomics data differentiates the data according to the ER status (C), and By using neural networks, the metabolic data can be reduced to the transcriptomics embedding space, now differentiating the ER status.

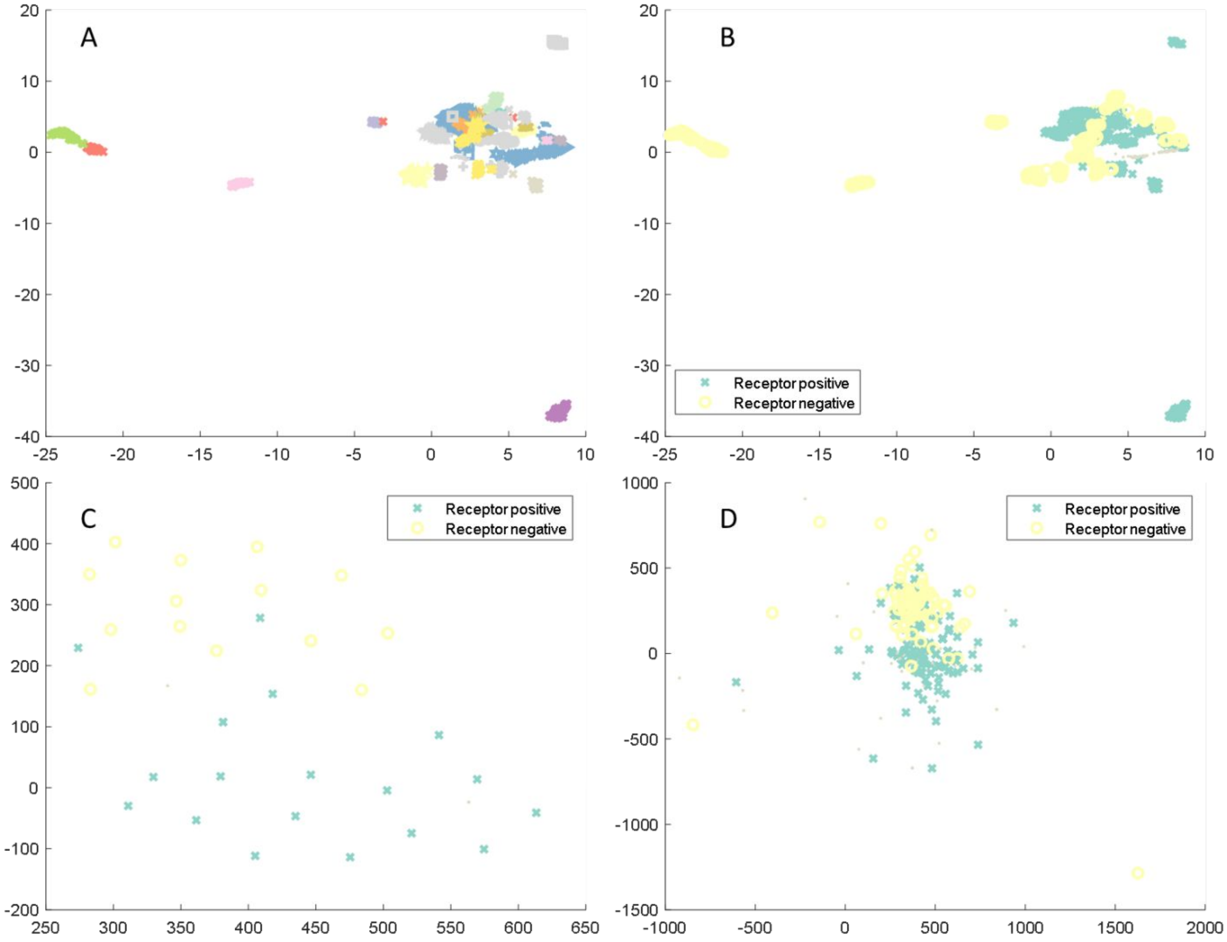

Figure S6. A comparison of ordered vs. random subsampling on the accuracy of the segmentation as evaluated by comparing the correlation to the t-SNE results. This shows no difference in these two sampling methods.

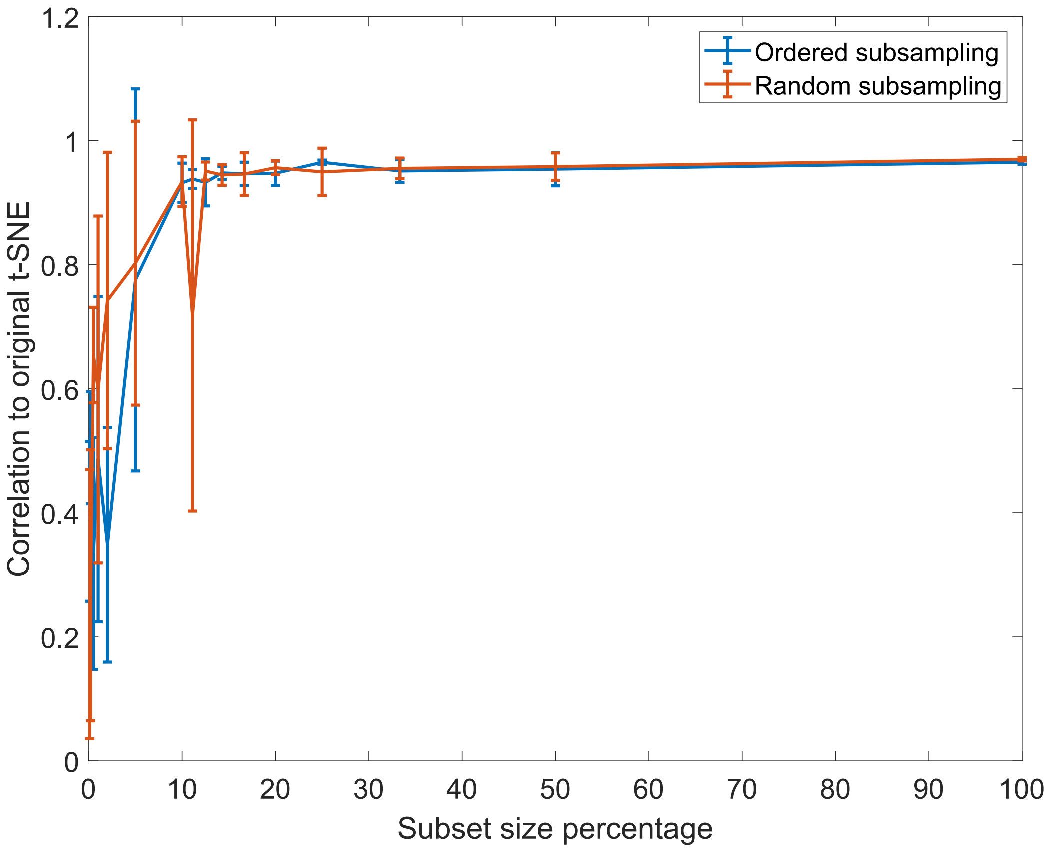

Figure S7. Comparison of the random and ordered subsampling on the resulting images from the sagittal brain dataset. These results show no major differences in the anatomical features obtained using these two different sampling approaches.

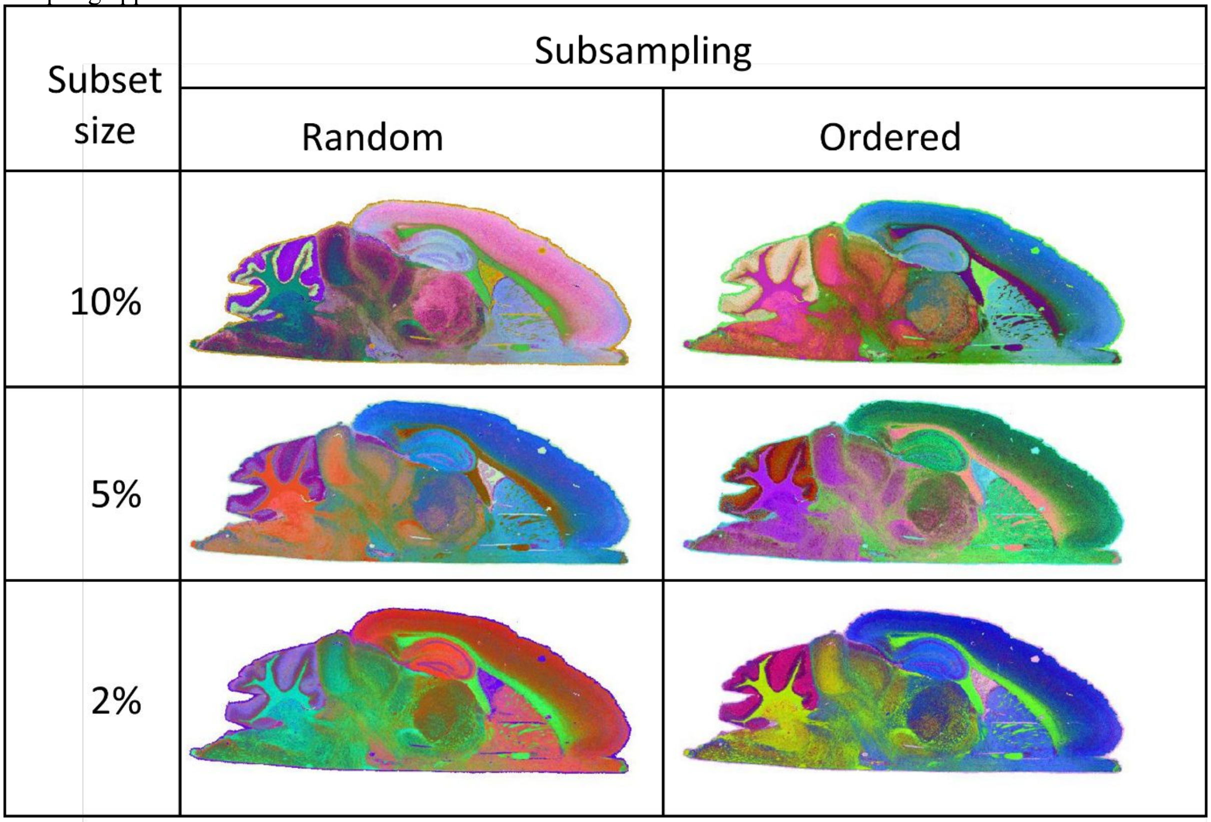

Figure S8. Comparison of the t-SNE embedding on the data from Figure 5, showing the whole data embedding vs. the embedding from just the subset. The subset of data occupies a similar space to the final embedding, which indicates that the subsampling in this instance has effectively captured the full range of data within the whole data

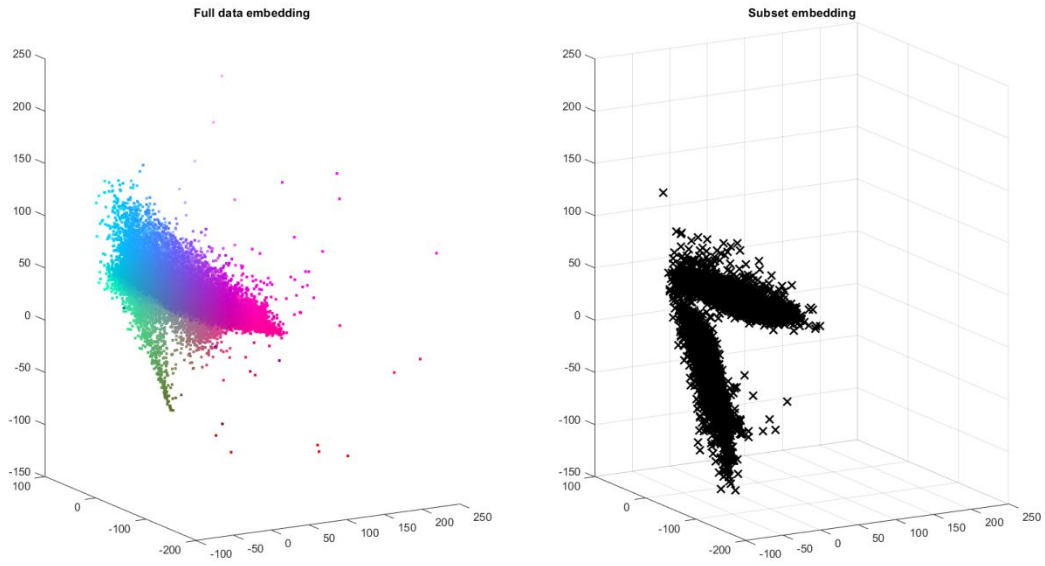

Figure S9 Neural network t-SNE (top), and NN UMAP (bottom) performed with different t-SNE and UMAP hyperparamaters (perplexity 1000, exaggeration 1, and nearest neighbours 100, minimum distance 0.1), showing good anatomical segmentation remains when these parameters are altered. The bottom plot shows the embedding scores (each dimension plotted in its corresponding colour) for the UMAP reduction against the matching neural network trained embedding.

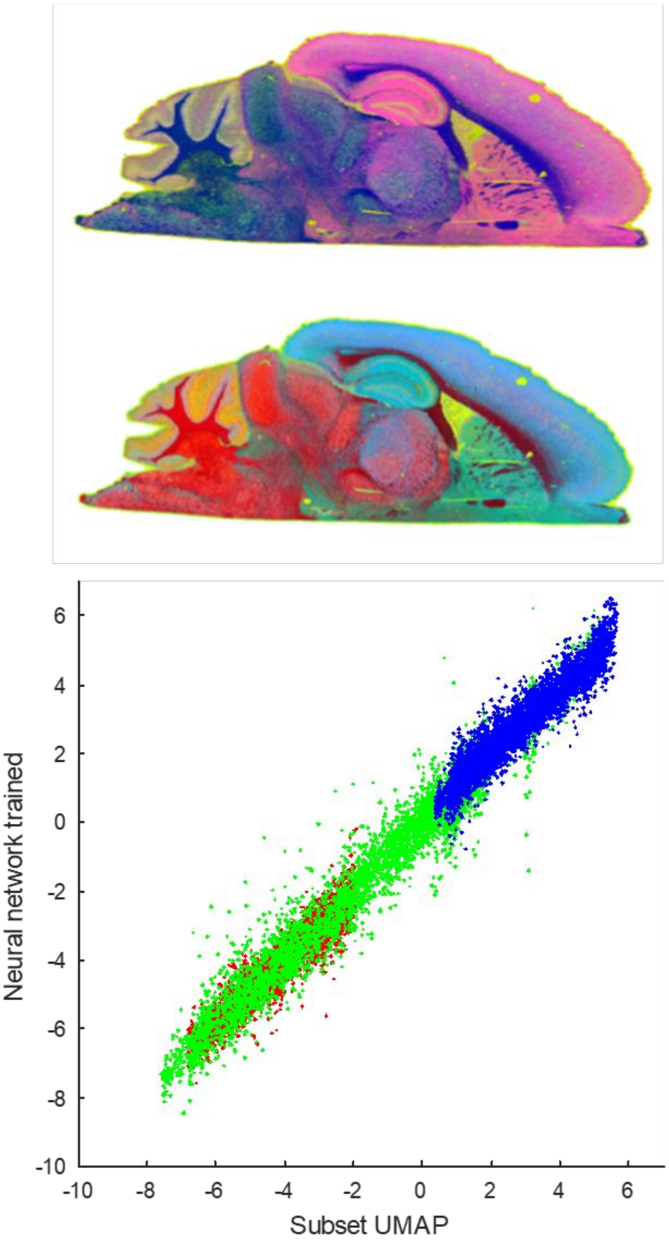

Figure S10. Comparison of the NN-tSNE embedding on the data when t-SNE is performed on different sized subsets. The threshold at which the anatomical feature segmentation is dependent on the number of pixels rather than the percentage of the original data size.

Subset %	Coronal		Transverse		Sagittal	
	Images	Pixels	Images	Pixels	Images	Pixels
100	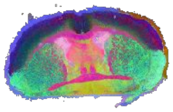	29554	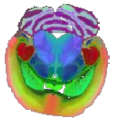	9295	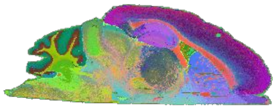	123557
10	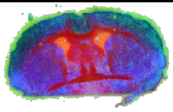	2955	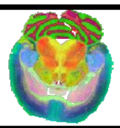	930	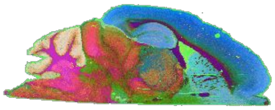	12356
5	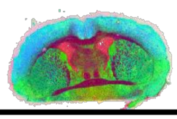	1478	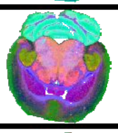	465	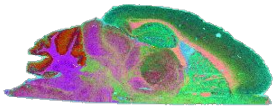	6178
2	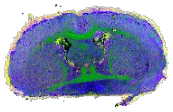	591	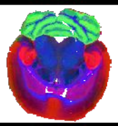	186	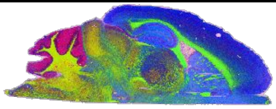	2471
1	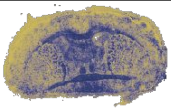	296	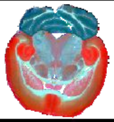	93	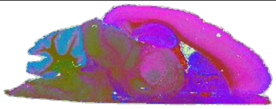	1236
0.5	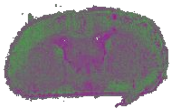	148	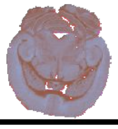	46	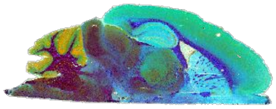	618
0.2	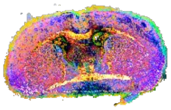	59	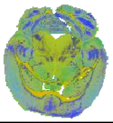	19	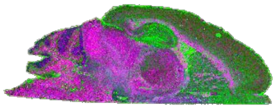	247
0.1	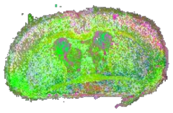	30	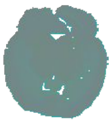	9	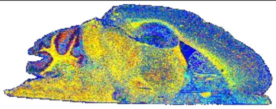	124

Figure S11. Autocorrelation applied to measure the influence of subset size on the image quality of the NN-tSNE results on the sagittal mouse brain data.

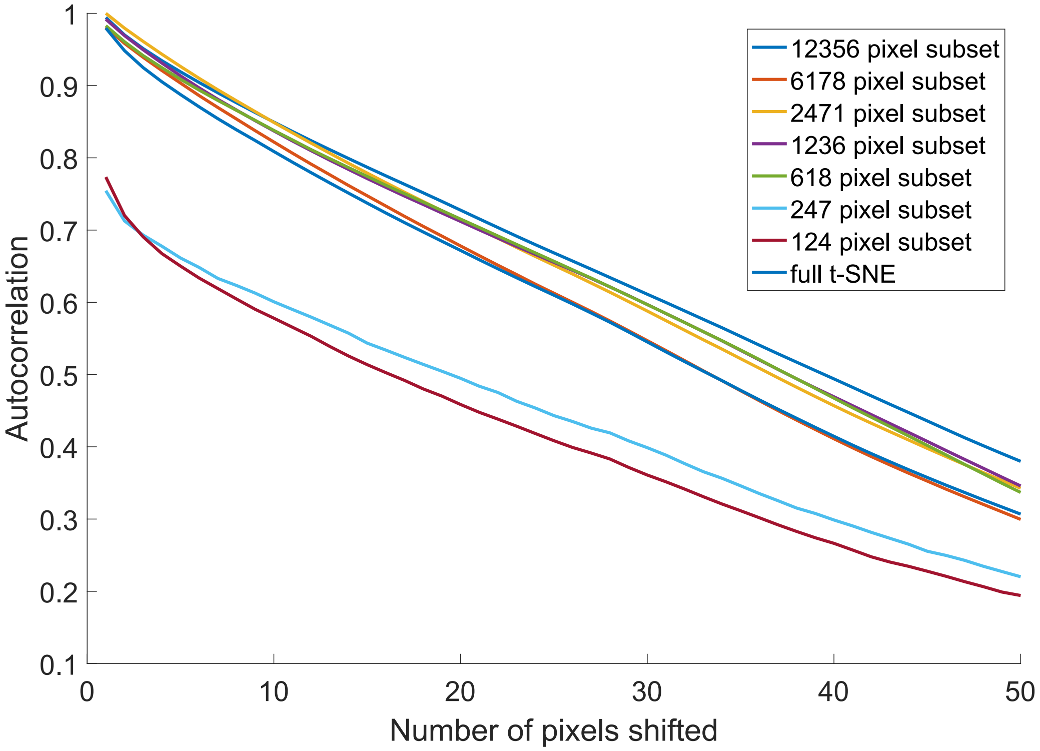

Figure S12. Comparison of the time taken to perform t-SNE and neural network training on different sized datasets. Above 10,000 pixels this becomes prohibitively slow to run as a routine analysis, whereas by preforming NN-tSNE, this can be reduced to a much more feasible timeframe. Of note, to run NN t-SNE on over 10,000 pixels you would still only need to train the data using 2,000 pixels (taking around 3 minutes).

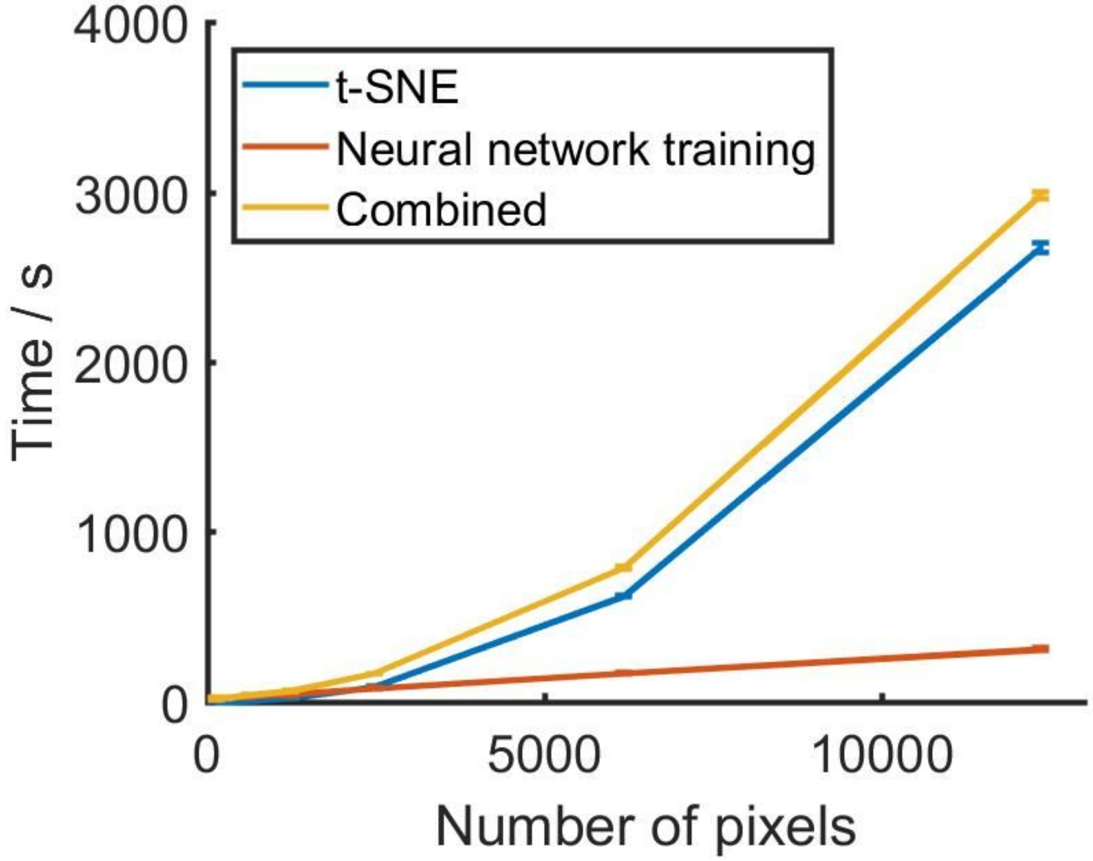

Figure S13. Comparison of the RAM requirement for the neural network training step compared to the t-SNE embedding of the subset. The t-SNE requires significantly more RAM than the neural network training.

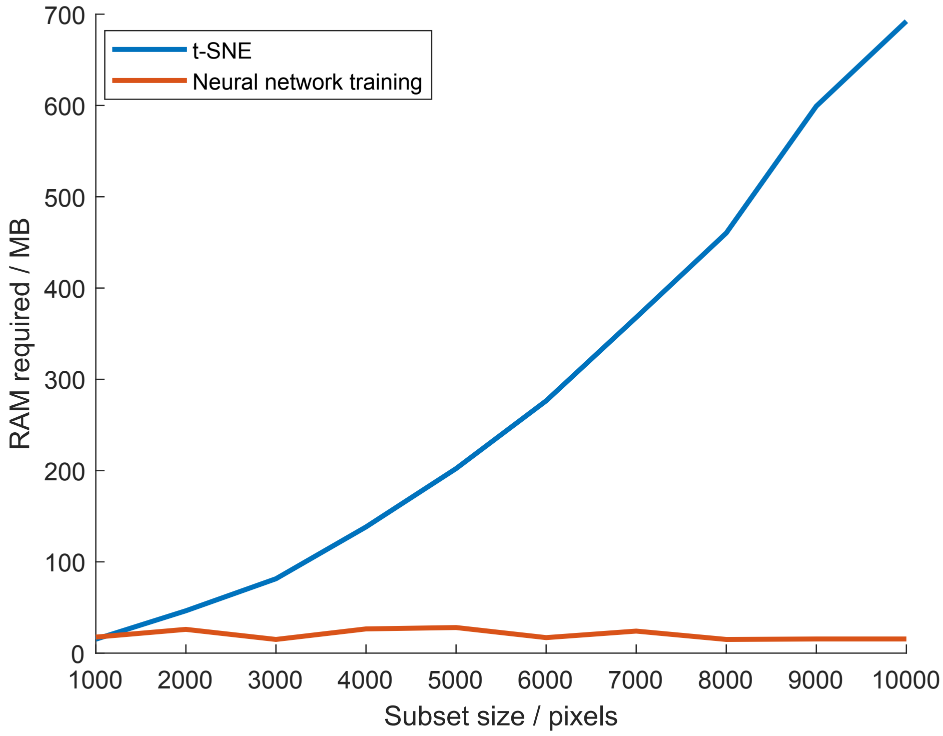

Figure S14. Comparison of the returned spectral contribution from an actual spectrum, as compared to their original spectra. Each one shows a high correlation to the spectrum that it was derived from.

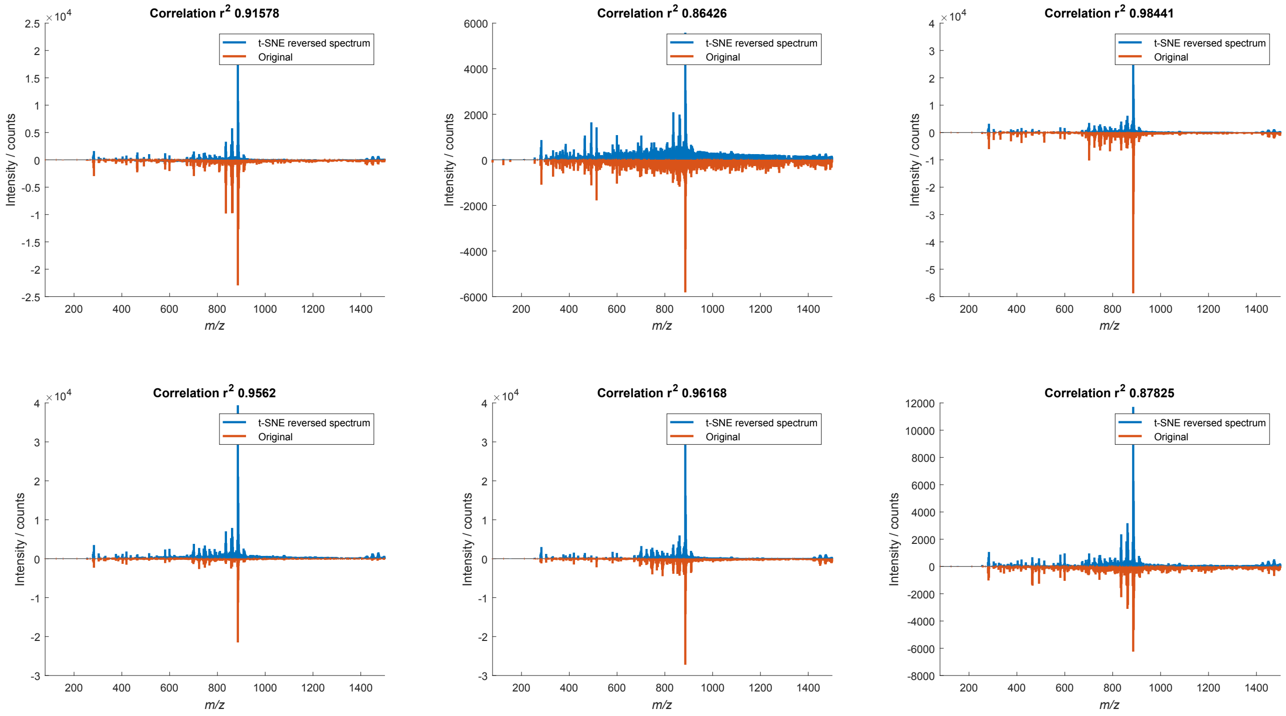

Figure S15. Comparison of the different neural network training methods applied to the MSI image of sagittal mouse brain evaluated on the correlation to the original t-SNE and the anatomical feature identification. The Levenberg-Marquardt and Bayesian regularisation methods are far superior to all other training approaches.

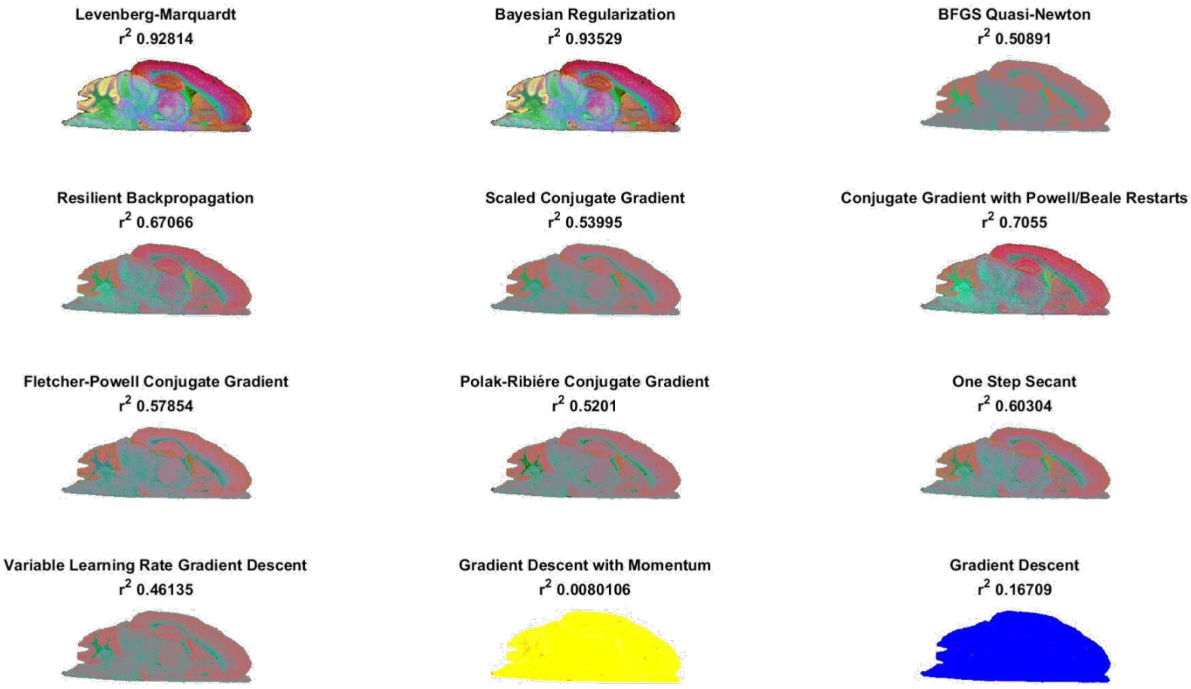

5
